# 16 Years of breed management brings substantial improvement in population genetics of the endangered Cleveland Bay Horse

**DOI:** 10.1002/ece3.8118

**Published:** 2021-10-03

**Authors:** Andrew Dell, Mark Curry, Elena Hunter, Ruth Dalton, Kelly Yarnell, Gareth Starbuck, Philippe B. Wilson

**Affiliations:** ^1^ Department of Biological Sciences University of Lincoln Lincoln UK; ^2^ School of Animal, Rural and Environmental Sciences Nottingham Trent University, Brackenhurst Campus Southwell UK; ^3^ Whinpot Farm Kendal UK

**Keywords:** cleveland bay, effective population size, population genetics

## Abstract

The consequences of poor breed management and inbreeding can range from gradual declines in individual productivity to more serious fertility and mortality concerns. However, many small and closed groups, as well as larger unmanaged populations, are plagued by genetic regression, often due to a dearth in breeding support tools which are accessible and easy to use in supporting decision‐making. To address this, we have developed a population management tool (BCAS, Breed Conservation and Management System) based on individual relatedness assessed using pedigree‐based kinship, which offers breeding recommendations for such populations. Moreover, we demonstrate the success of this tool in 16 years of employment in a closed equine population native to the UK, most notably, the rate of inbreeding reducing from more than 3% per generation, to less than 0.5%, or that attributed to genetic drift, as assessed over the last 16 years of implementation. Furthermore, with adherence to this program, the long‐term impact of poor management has been reversed and the genetic resource within the breed has grown from an effective population size of 20 in 1994 to more than 140 in 2020. The development and availability of our BCAS for breed management and selection establish a new paradigm for the successful maintenance of genetic resources in animal populations.

## Graphical abstract

Across the last 16 years, a genetic breed management program has led to the British population of the Cleveland Bay transitioning from an effective population size well below the UN FAO limit of concern of 50, to a stratified breeding system with *N*_e_ now over 145.

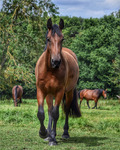

## 1 INTRODUCTION

The need to manage inbreeding in closed populations of animals such as domestic pets, captive populations of wildlife, or farmed livestock has been further emerging in international policy through individual national efforts, as well as guidance from regulatory bodies such as the United Nations Farm Animal Organization. As gene sequencing technologies become more widespread and levels of inbreeding can now be assessed using runs of homozygosity (ROH) determined using single‐nucleotide polymorphisms (SNPs), it has been suggested that pedigrees alone are no longer adequate to formulate breed management programs (Dell et al., 2020a).

Conversely, it has been suggested that where a pedigree is deep, it may well remain the preferred tool to assist in formulating breed management programs to control the rate of increase in inbreeding (Dell et al., 2020b), maximize effective population size (*N*_e_), and limit the expression of deleterious alleles (Dell et al., 2020a, 2020b). In this article, we report a “real world,” long‐term, pedigree‐based breed management scheme, in which a globally endangered equine breed, with a robust and deep (>36 generation) pedigree, has seen an effective population size increase from unviable levels, well below the threshold set by the Food and Agriculture Organization of the United Nations (FAO) to levels where the breed has a much more secure future.

**FIGURE 1:**
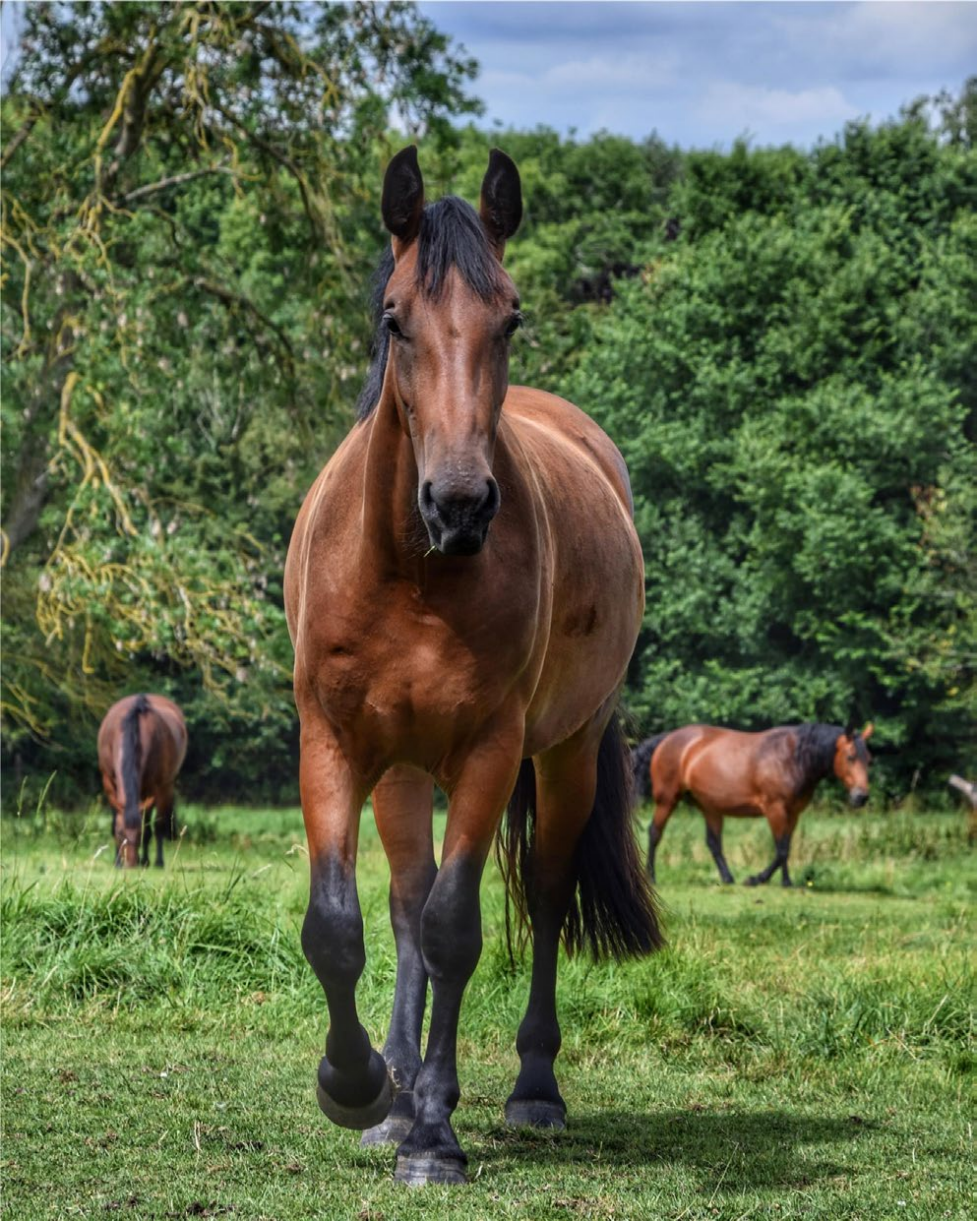
Cleveland Bay Scarholme Litha Photo credit: David Snooks

The Cleveland Bay Horse (Figure 1) is a heritage British breed that originated three centuries ago in the northeast of England where it was used both as a carriage horse and to work the land. Its origins in the matriline are said to derive from the Chapman horse, the packhorse of Tudor England (Dent, 1978).

Recent studies have confirmed that the breed's paternal origins come from imported Turkoman horses (Khanshour et al., 2019) which were also among the founders of the modern Thoroughbred.

The breed is one of seven equine breeds listed as priority (restricted effective population size) by the Rare Breeds Survival Trust, and previous studies (Emmerson, 1984; Walling, 1994) have shown that inbreeding in this essentially closed population may have been as high as 22%. Microsatellite short tandem repeat (STR) analysis has shown that the breed has the third lowest level of variation of any studied equine breed after the Clydesdale and Friesian (Khanshour et al., 2019).

The Cleveland Bay Horse Society (CBHS) published its first studbook in 1885, containing retrospective pedigrees of animals dating back to 1723 (Emmerson, 1984). This now yields a non‐Thoroughbred studbook dating back almost 300 years and covering 38 generations. Genetic and demographic analyses reported in previous studies (Dell et al., 2020b; Walling, 1994) suggest that even at the time of formation of the Society in 1884, the genetic resources within the breed were secure. Many founder animals were no longer represented in the then living population, and other lines were lost soon afterward. Although Wright had yet to formulate the concept of the inbreeding coefficient (Wright, 1922), it is evident that lack of breed management was leading to its disproportionate accumulation. As a consequence, *N*_e_ was restricted and well below the threshold of 50 that is now deemed necessary for the maintenance of the genetic health of the breed (FAO I, 2004).

In the ensuing 135 years, the breed has suffered a substantial decline in numbers due to modernization of transport infrastructure and mechanization of agriculture, as well as substantial losses in two world wars. By the 1950s, the breed was close to extinction, with only four pure bred stallions remaining.

The efforts of a small number of dedicated breeders including HM the Queen brought the breed back from the brink of extinction (Vila et al., 2001), and in 1971, the Cleveland Bay was one of the original equine breeds recognized as being endangered by the newly founded Rare Breeds Survival Trust.

The focus of this study is to report on our development of the knowledge base surrounding the genetic status of the Cleveland Bay Horse gained from pedigree analysis and which has been used to design and implement a breed management strategy, which is both scientifically appropriate and practically sustainable in managing the endangered global population.

This program, based on managing inbreeding through control of mean kinships determined from pedigree analysis, first implemented by the Cleveland Bay Horse Society in 2004, has been computed and adhered to for 17 years which approaches two complete generations and provides a model not only for breeders of other endangered equine breeds but also for other types of livestock and domestic animals to demonstrate the effectiveness of such a breed management strategy in vivo and in practice.

Indeed, practically and throughout this study, we use the Cleveland Bay horse as a case study for a genetically fragile population, which our Breed Conservation and Analysis System (BCAS) has been implemented to support and reverse the decline in genetic diversity within the population. The BCAS is implemented within the Cloud‐Lines online framework (https://cloud‐lines.com). We successfully demonstrated this application over more than 16 years of management, yielding an *N*_e_ now over 140. Furthermore, the theory and application listed herein remain widely applicable to at‐risk and endangered populations in their management, and we offer perspective supporting these efforts.

**FIGURE 2:**
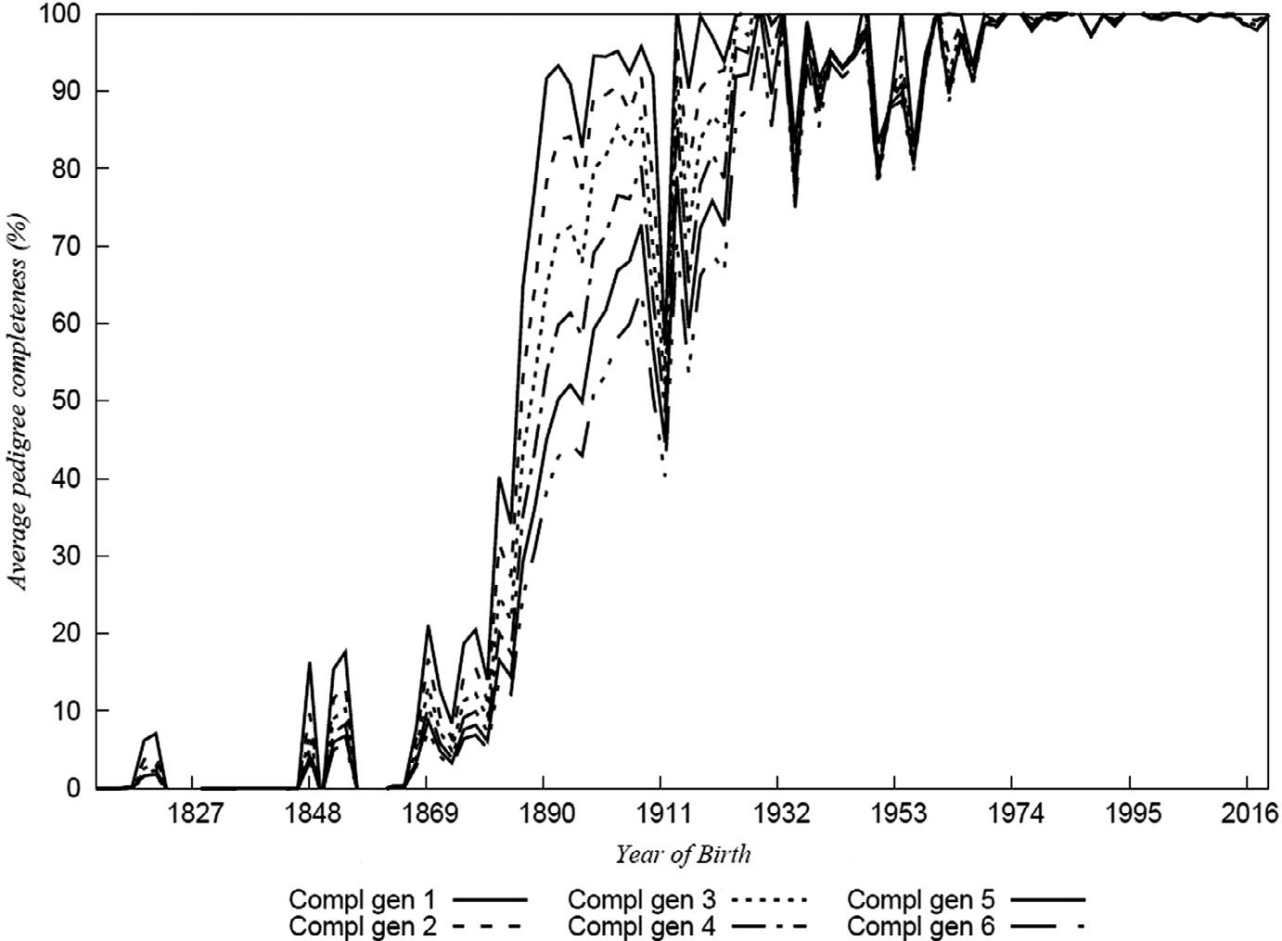
Pedigree completeness of the Cleveland Bay Studbook 1800–2020 for 1–6 generations

Previously reported evaluation of the studbook (Dell et al., 2020b) has shown that the Cleveland Bay horse has a very deep and robust studbook extending back over 36 generations. Evaluation using PopRep (Groeneveld et al., 2009) demonstrates that in the 2015 foaling year, the pedigrees of every animal was complete to six generations, only reducing to 99.8% of animals at six generations in 2020 when a limited number of females were admitted to the studbook via a grading up scheme (Figure 2).

### 1.1 Past breeding practice

From its origin in northeastern England, the Cleveland Bay horse was very much a horse for the general population. It was kept by hill farmers to work the land and tend their flocks, by landed gentry to breed carriage horses, and by royalty (Scarth‐Dixon, 1929). The pattern of breeding carried out by this diverse group of supporters has been as wide and varied as their demographics themselves.

Arguably, it is the lone farmer who perhaps kept only very small numbers of mares for breeding or who would use one of the stallions that travelled throughout the region, providing their commercial services. The 18th and 19th centuries also saw the northeast of England become home to many of the early Thoroughbred horses. There is now substantial evidence that supports the suggestion that many Cleveland mares were bred to such stallions and introgression of “Thoroughbred blood” into the breed occurred at this time (Pease, 1935; Scarth‐Dixon, 1929).

Further, the large studs such as those maintained on the Cholderton Estate in Wiltshire and at Hampton Court (by King George V) on the western outskirts of London employed one or more stallions in order to cover relatively large groups of mares. At Hampton Court, animals were bred for service at the Royal Mews, whereas at Cholderton, horses were bred both for work on the estate and sold as a source of revenue. Many animals were sent for export, including to the USA (Pease, 1935).

At a time when the restricted gene pool meant that finding outcrosses was becoming almost impossible, the CBHS was responsible for importing the stallion Farnley Exchange back into the UK from the USA (Emmerson, 1984). Now, many of the large studs have disappeared. However, the Cholderton Estate remains one of the major Cleveland Bay breeders in the UK, and HM the Queen continues to produce Cleveland Bay horses under the Hampton Court prefix.

Currently, the majority of actively bred Cleveland Bay mares are owned by individuals who kept them as a hobby in small numbers for their rare breed status. Many breeders had been using stallions chosen not necessarily because of genetic suitability but because of geographic proximity, although the increasing use of artificial insemination (AI) in the USA, Canada, and Australia has broadened the choice of stallions available. However, at the turn of the millennium, it was clear that in the absence of a coordinated breeding program or management system, inbreeding was accumulating at a rate higher than would be expected purely because of the limited population size and closed nature of the studbook. This had already been highlighted when the results of an undergraduate dissertation study were published in Volume 33 of the studbook (Walling, 1994). This information gave rise to a number of breed conferences at which the need for a proactive breed management plan was identified.

Although the need for management to prevent the continued loss of diversity was highlighted, what was not clear was the manner in which this would deliver a real‐world impact to support the genetic resources within the breed. The difficulties being experienced by the Cleveland Bay population as an endangered breed had more in common with those experienced in the field of wildlife and captive animal conservation than livestock breeding because of the limited number of animals of breeding age, the limited number of founders, and the loss of founder representation (Dell et al., 2020b). However, computer systems being used by zoological parks had not proven suitable in the management of equine breeds (Hall, 2004).

### 1.2 Breed management theory

The current recommendation of the United Nations Farm Animal Organization (FAO) is to maintain breeds with a maximum rate of accumulation of inbreeding of 1% per generation (FAO I, 2004). In order to do this, it is necessary to maintain a minimum *N*_e_ of 50 animals (the rate of inbreeding Δ*F* = 1/2*N*_e_). Numerous systems of managing the diversity of livestock and wildlife populations have been developed in recent years, and in order to understand the system that was determined to be the most appropriate for the Cleveland Bay horse, it is timely to review those available at the inception of the BCAS framework.

The identification of individuals or populations that are the most important contributors to genetic diversity requires quantitative assessment. Typically, all methods of quantitative assessment of diversity can be described by one of two methods: the Weitzman diversity method and the core set diversity method (Eding et al., 2002). The Weitzman method of determining diversity uses both genetic and nongenetic information, and a recursive algorithm to calculate the relative contributions of breeds or individuals to total population genetic diversity, based on genetic distance (Weitzman, 1992). However, despite its widespread use in the management of livestock populations, there is growing criticism of the Weitzman approach as it only accounts for between‐breed diversity and neglects within‐breed diversity (Caballero & Toro, 2002; Eding et al., 2002).

The concept of core sets was first proposed in the field of plant breeding and was defined as the minimum set of lines or types of a plant species that would still represent the genetic diversity of that species (Eding & Meuwissen, 2001). The aim of the core set model is to eliminate the genetic overlap between each of these lines. The genetic similarity or overlap between individuals or populations can be described using a coefficient of kinship. This was first defined by Malècot as the probability that two alleles, taken at random from two individuals, are identical by descent (Falconer & MacKay, 1996; Frankham et al., 2002; Malècot, 1948). The coefficient of kinship describes genetic diversity in terms of alleles (Caballero & Toro, 2002) and also in terms of quantitative genetic variation, without requiring a detailed knowledge of the genetic processes involved (Eding & Meuwissen, 2001). Minimizing the genetic overlap is equivalent to minimizing kinship in a set of breeds by adjusting contributions of each population or individual to the core set (Eding et al., 2002).

The kinship coefficient carries an analogous relationship with other important measures of relatedness, in particular the inbreeding coefficient (Wright, 1922). This is described as the probability that two alleles at one locus in an individual are identical by descent. It is thus equivalent to the coefficient of kinship of the parents.

Wright (1922) also defined the relationship coefficient. There is a direct relationship between the relationship coefficient and the kinship coefficient best expressed as follows:

(1) $$R_{\mathrm{st}}=2K_{\mathrm{st}},$$

where *s* and *t* are two individuals, *R* is the relationship coefficient between them, and *K* is their kinship coefficient.

Calculation of both the kinship coefficient and relationship coefficient is straightforward where complete pedigree information is available (Flury et al., 2006). They can be computed manually using the numerator relationship matrix method (Emik & Terrill, 1949). Computer algorithms make the calculation of both coefficients a much less labor‐intensive process, particularly where large pedigrees are concerned. At the time, the CBHS breeding program was being established in the early 2000s, the GENES software (Lacy, 1998) distributed as part of the Single Population Analysis and Record Keeping System (SPARKS) by the umbrella organization for all captive wildlife management programs. ISIS (now Species 360) was the only readily available software capable of computing both coefficients efficiently and effectively.

Having determined the kinship of any individual in a population to all other members, it is possible to calculate a mean kinship. This is the average of the kinship coefficients between a single animal and all of the other candidates (currently living and fertile animals) including itself. It can be expressed by the following equation:

(2) $${\text{mk}}_{i}=\frac{1}{N}\sum_{j=1}^{N}f_{\mathrm{ij}},$$

where *N* is the number of candidates in the population and *ƒ_ij_* is the kinship between individual *i* and individual *j*. Each individual thus has its own mean kinship (mk) and its own kinship coefficient (*k*) to every other individual in the population. Individuals with a low mean kinship are genetically important animals within a population. The mean kinship is dependent on the whole population, and so the mean kinship of any individual can change over time as the population changes. The goal of prioritizing animals having a low mean kinship is to lower the average mean kinship of the whole population. This is the arithmetic mean of all the individual mean kinships, calculated as follows:

(3) $$\bar{\text{mk}}=\frac{1}{N^{2}}\sum_{i=1}^{N}\sum_{j=1}^{N}f_{\mathrm{ij}}.$$

An individual whose mean kinship is lower than the population mean kinship will have fewer relatives in the population than an individual, where mk*_i_* > mk_pop_ (VanDyke, 2008).

The use of mean kinship as a management tool for captive populations of wild animals was proposed by Ballou and Lacy in 1994 and has been universally adopted by zoological collections throughout the world as the system of choice for advising population management plans. They compared three genetic management strategies: the maximum avoidance of inbreeding (MAI), random mating, and management by minimizing kinship. They found that mean kinship performed significantly better than all other strategies (Ballou & Lacy, 1995). The same breeding strategies were evaluated by theoretical modeling and in the laboratory with *Drosophila* fruit flies, and the same conclusions were reached (Montgomery et al., 1997). Mean kinship analysis was found to provide rationale for an efficient and relatively simple strategy for the maintenance of expected heterozygosity and allele frequency in populations with complex pedigrees. The strategy equates to those which maximize founder genome equivalents and gene diversity as well as balancing founder contributions (Ballou et al., 1995; Lacy, 1989).

Although management of captive wildlife populations by mean kinship is now universally adopted by zoological associations across the globe, until now, there were significant difficulties that prevent the system being adopted for the management of livestock biodiversity (Hall, 2004). The main issues appear to be the multitude of breeding practices carried out on different livestock species, the geographic distribution, and the scale of breeding populations, which limit the exchange of breeding animals in farming operations. Furthermore, the unequal genetic contributions of males and females in many breeding programs acts as a further confounding factor. It is usual for only a limited number of sires to be kept in most livestock breeding programs, with a disproportionate contribution of selected individuals to the genome. A classic example of this is seen in Holstein cattle breeding, where the global population is in hundreds of thousands, but because of unequal contributions of parent animals, the limited number of sires, and the influence of artificial insemination, the effective population size is <50 (Mrode et al., 2009). Additional problems arise when dealing with the demographic makeup of the population. Much of the original mathematical modeling of management by mean kinships was based on the premise of discrete generations. In practice, this is seldom the case in livestock populations, where overlapping generations are common. Although the Weitzman approach had a significant influence on livestock conservation and management in the early 1990s, it has been displaced as the system of choice by that of optimum genetic contributions (Meuwissen, 1997).

Two prime decisions drive most breeding programs, namely, (a) selection decisions based on the choice of animals which should be used for breeding and how widely they should be used and (b) mating decisions, concerned with how the selected animals should be mated. Selection strategies include increasing the number selected, restricting the number selected per individual family, and reducing the emphasis given to family information (Toro et al., 1988; Villanueva et al., 1994). Work on mating decisions has included factorial design, minimum coancestry mating and compensatory matings (Caballero et al., 1996).

Although these methods have been successful, they consider rates of genetic gain and inbreeding separately, and although they do control the rate of inbreeding, they also lead to losses in selection response (Villanueva et al., 2004). Meuwissen (1997) and Grundy et al. (1998) produced and refined a system of optimized selection, in which Δ*F* could be managed without any loss in genetic gain (Grundy et al., 1998; Meuwissen, 1997).

Optimal contribution selection (OCS) is a strategy that calculates contributions per candidate animal (fertile individuals) such that the weighted average mean kinship is minimized. Average mean kinship among candidates is expressed by the following equation:

(4) $$\bar{\text{mk}}={c^{\prime }}_{\text{EC}}{\mathrm{Fc}}_{\text{EC}},$$

where **F** is a matrix of kinships between all candidates, including self‐kinship, and **c**_EC_ is a column vector of equal contributions for each candidate in the next generation (Meuwissen, 1997). The sum of all elements of **c**_EC_ will equal 1. Average mean kinship and thus mk in future generations can be decreased or increased by varying the contributions of candidates. By finding an optimum contribution vector **c**_OC_ that minimizes **c**_Fc_, it is possible to minimize average mean kinship (Eding & Meuwissen, 2003; Meuwissen, 1997):

(5) $$c_{\text{OC}}=\frac{F^{‐1}1}{1^{\prime }F^{‐1}1}.$$

In theory, OCS is the most efficient method to minimize kinship (Pong‐Wong & Woolliams, 2007; Sonesson & Meuwissen, 2001). Application of optimum selection requires only basic information that is usually available to breeding programs, such as pedigree information and estimated breeding value (EBV). Estimated breeding values are typically calculated using best linear unbiased prediction (BLUP) methods (Henderson, 1985).

The use of BLUP systems is now widespread in the management of livestock populations. They involve scoring for both genetic and phenotypic information. This can involve single or multiple traits. In equine management, BLUP has been adopted to guide the breeding of thoroughbred racehorses, Swedish trotters, and Icelandic ponies. There is however growing criticism of the system for genetic conservation of rarer breeds as the probability of co‐selecting related animals is high as the estimates produced are more highly correlated than true breeding values. This results in higher rates of inbreeding in the population (Sonesson, 2002).

Various software tools for guiding breeding programs based on optimum contributions have been developed. The first was GENCONT (Sonesson & Meuwissen, 2000) which has been criticized for the limitation of being modeled upon a pattern of discrete generations, which is unlikely in real‐time livestock production (Sonesson & Meuwissen, 2002). Further developments have been incorporated to overcome these shortcomings, and EVA (Berg et al., 2007) and OPTISEL (Wellmann, 2019) have become the systems of choice for managing optimum genetic contributions. Both systems enable control of the rate of inbreeding while managing genetic gain, by incorporating estimated breeding values. The latter is most often obtained through BLUP but could equally arise from a score such as Genetic Conservation Index (Alderson, 1992).

Having determined the dearth of solutions appropriate for advising a breed management scheme for the Cleveland Bay horse, it became clear that a custom scheme would need to be designed that would (a) incorporate current scientific advice and methods; (b) present advisory information in a way that was acceptable to breeders, and (c) provide a user‐friendly solution to the two‐stage problem of both parental selection and determining the genetic contribution of parents to the next generation. The criterion that is recommended to generate the least amount of inbreeding and greatest genetic gain is employing minimum coancestry matings (Caballero et al., 1996; Meuwissen, 2009).

The inbreeding coefficient of an individual will depend on the amount of common ancestry in its two parents. When considering the inbreeding in the progeny, we can consider the degree of relationship by descent of the two parent animals. This is the coancestry or kinship coefficient (Falconer & MacKay, 1996).

Minimum coancestry matings delay the onset of inbreeding by minimizing it in the next generation (Caballero et al., 1996; Sonesson & Meuwissen, 2001; Toro et al., 1988). Second, after the onset of inbreeding, minimum coancestry has been shown to generate lower rates of inbreeding when compared with alternatives (Caballero et al., 1996).

The basis for the minimum coancestry model lies in the definition of the genetic contribution of an ancestor being the proportion of genes that it contributes to the descendants of a population. After several generations, the contributions of all of the ancestors will stabilize, at which point they become referred to as long‐term genetic contributions. Once this is the case, an ancestor will make the same contribution to all descendants, with contributions differing between ancestors. The more ancestors that play a part in long‐term genetic contribution, the lower is the rate of inbreeding. Minimum coancestry mating disperses the contributions more rapidly across the population and increases the number of ancestors that contribute to each descendent (Villanueva et al., 2004).

Although work to improve on the minimum coancestry model continued in the shape of minimizing the covariance of ancestral contributions (MCAC mating) (Henryon et al., 2009), it was decided that a breed advisory scheme which selected parents of similar mean kinship and also minimized coancestry of the progeny would be most suitable for the Cleveland Bay horse.

## 2 MATERIALS AND METHODS

In 1994, the Cleveland Bay Horse Society published part of an undergraduate research thesis in Volume 33 of its studbook. That work, “An Analysis of The Breed Structure of The Cleveland Bay Horse And A Plan For The Maximum Maintenance Of The Genome” (Walling, 1994) highlighted the accumulation of inbreeding and the unequal contribution of sires and suggested some specific matings based on minimum coancestry. It went on to suggest that the implementation of a breed management scheme should be a priority for the society.

The Walling study produced much debate among breeders and in the Council of the CBHS, but it was the catalyst for a series of breed conferences. It also provided momentum for digitization of the entire studbook, and the resulting electronic database now containing in excess of 6,000 records has proven a valuable tool for more detailed analysis (Dell et al., 2020b).

Having confirmed the previously reported high levels of inbreeding and loss of founder representation, the consensus among both the Council of the CBHS and breeders was that a breed management solution should be implemented. However, investigation showed there were no appropriate user‐ and stakeholder‐friendly options to undertake this. Early private use of the GENES software (Lacy, 1998) had shown it capable of calculating both kinships and mean kinships and the necessary relationship matrix of coancestry between all possible parents. This information had been used for a number of years to advise individual Cleveland Bay breeders on mate selection and minimizing inbreeding. After formal discussion, the CBHS decided to adopt the SPARKS (Single Population and Animal Record Keeping System) software that was being used in zoos around the world to advise on captive wildlife management (Earnheardt, 1999).

The entire Cleveland Bay Studbook was entered into SPARKS (Earnheardt, 1999) with assistance from staff at ISIS (now Species 360). This included rewriting an extended version of the software to deal with the large number of animals in the studbook. At the time it was first established in 2004, the Cleveland Bay SPARKS database was the largest such dataset in the world, the software having been designed for the management of much smaller populations of captive wildlife.

### 2.1 Scheme aims and design

Any breed management scheme involving domestic horses is likely to be a long‐term project due of the large generation interval involved. The average generation interval of the Cleveland Bay Horse has been determined as 9 years (Dell et al., 2020b). It was important to design a scheme that the majority of breeders could adopt, feeling that it met not only the needs of global population management but also offering individual breeder choice.

A mating scheme based on controlling the rate of inbreeding by minimizing coancestry and selecting mating pairs by mean kinship requires two fundamental pieces of information: (a) the mean kinship of each animal in the population and (b) the coancestry coefficient (theoretical inbreeding coefficient) of all possible male and female pairings. These were originally calculated using GENES (Lacy, 1998).

In order to subdivide the population and with a view to advising the mating of animals of similar mean kinship, all living animals were allocated to one of seven “bands” based on mean kinship values. The allocation of these bands and number of horses in each at the outset of the project in 2004 is shown in Table 1.

**TABLE 1:** Mean kinship band allocation

Designated band	Mean kinship (minimum)	Mean kinship (maximum)	Number of horses in band (*N*)
A	0	0.19	6
B	0.19	0.2	19
C	0.2	0.21	65
D	0.21	0.22	190
E	0.22	0.23	364
F	0.23	0.24	386
G	0.24	1	40

**FIGURE 3:**
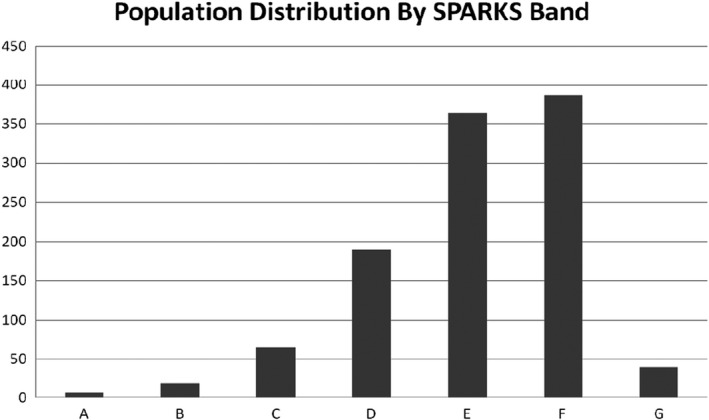
Population distribution by SPARKS (mean kinship) band

Figure 3 shows the distribution of this population across these seven bands. The few members in the lower bands A and B are those animals least related to the whole population, and the majority of the population are placed in bands E and F, being more closely related to the majority of the population.

Although animals in the lower bands will have a higher priority for breeding, it is important that they are not mated with animals from the upper bands. Indeed, as well as increasing the frequency of the less common alleles carried by animals of lower mean kinship, such matings will also increase the frequency of the more common alleles carried by members of the higher bands. Once brought together in such matings, the two sets become inseparable, and it is no longer possible to increase the frequency of one without the other (Ballou & Lacy, 1995).

It was also necessary to collate the coancestry coefficient of every possible pairing of male and female animals in order to establish the breed management scheme. This is equivalent to the inbreeding coefficient of the foal that would be produced by that mating and will be carried by that animal for its lifetime, irrespective of additions to the population by other breeding or natural losses. GENES was also used to calculate a matrix of all breeding pairs based on every animal in the population. Although the calculation of the coancestry matrix was found to be straightforward, the format in which the results are presented by the software was found to carry user tolerance issues. The broken tabulation lent itself neither to direct interpretation nor easily to conversion into a more acceptable format.

To advise individual breeders on the suitability of one or two possible matings using the data in the existing format was found to be both time‐consuming and not practical in the long term and with a high throughput of data. It became clear that in order to become a viable scheme, the information would need to be presented in a much more user‐friendly format. To achieve this, registration, mean kinship, and coancestry data were imported into Filemaker™ to create a relational database.

Early on in the process of the construction of the scheme, it became clear that the data could be presented in two main ways, these being every possible mating for a selected female and every possible mating for a selected stallion. Although the use of the data in either way would clearly be of value in controlling the rate of inbreeding, discussion in the Council of the Cleveland Bay Horse Society identified the possibility of a stallion owner using male‐based data to proactively commercialize their stock and use the information for commercial advantage. The principal of breeding from as many males and females as possible is well‐established as it addresses the potentially damaging effect of using only a small number of stallions. This has a negative effect on census effective population size (*N*_e_) determined from the number of breeding males and females. In view of this, a CBHS Council decision was made only to support the scheme if it were restricted to providing mare‐based data. In this format, a mare owner would be able to see how compatible each of the licensed stallions would be with his or her own animal(s).

The final layout of the mare datasheets (see Appendix S1) contained data for every living mare of potential breeding age‐matched against every licensed stallion, together with the stallion location and availability by natural cover or artificial insemination. As of 2020, a total of 368 mares and 101 stallions are included in these datasheets. Of these stallions, 43 are in the UK, 36 overseas, and 22 are deceased but with frozen gametes still available. This is a considerable improvement on the stallion numbers available in the 1950s and 60s when only four stallions were standing in the UK.

By providing datasheets in the format shown, mating decisions remain firmly with the breeder and not the breed society. Including the suitability of every living licensed stallion, as well as the compatibility with deceased stallion where frozen semen remains available in GeneBank, enhances the possibility of every male as well as every female being used for breeding and contributing to the next generation which is a recognized priority in breeding schemes involving small populations (Oldenrboek et al., 2014).

The EU Zootechnical Regulations 2016 made it a legal requirement the breeding choice remains with individual breeders and not mandated by breed societies. The scheme described is by default fully compliant with that legislation.

The legislation and the need to breed from as many males and females as possible can be seen to be at odds with each other but, unlike in schemes based on optimum genetic contributions, no restrictions were placed on the number of times any one stallion could be used in a single breeding season. In practice, breeders make choices based on personal preference and geography, and this in association with advice against the overuse of any small group of stallions has been shown to be adequate to avoid compromising effective population size.

A copy of the datasheets and of the accompanying guidance notes are reproduced in detail in the Appendix S1. By 2018, the datasheets had evolved to incorporate a traffic light conditional formatting scheme to define four tiers of mating and make appropriate matings easier to identify. Those tiers based on mean kinship and coancestry are as follows:

Tier 1 (green)— Compliant and desirable in which animals of similar mean kinship (same or an adjacent band) producing progeny with coancestry lower than the mean kinship of the mare* (see footnote).
Tier 2 (yellow)—“Best of the rest”—parent animals from the same or adjacent bands with coancestry of the progeny greater than mean kinship of the mare but lower than the 0.24 threshold defining highly inbred (Tier 4) matings.
Tier 3 (orange)—“Discouraged”—matings of parent animals of dissimilar mean kinship (jumping bands) with coancestry of the progeny lower than the 0.24 threshold defining highly inbred matings.
Tier 4 (red)—“Avoid”—any mating with coancestry of progeny greater than the 0.24 threshold defining highly inbred matings.

*

In defining these tiers, a number of models were explored including avoiding matings with coancestry greater than the average inbreeding of the living population and including both parent animal mean kinship in the assessment. The final model was chosen to ensure that for the majority of mares there were a number of green and yellow Tier 1 and 2 matings available so that breeders were still presented with choices in stallion selection.

To accompany the datasheets, a set of guidance notes were produced to assist breeders in interpreting the data and making breeding decisions that would fit in with the overall objectives of the scheme (refer Appendix S1).

### 2.2 Scheme launch

The scheme was formally launched to Cleveland Bay breeders in April 2004, timed to take place at the start of the UK breeding year. Subsequent to its launch in the UK, the scheme was promoted to North American and Australasian breeders.

In the early years of the scheme, datasheets were distributed principally by paper copies mailed to breeders and electronic data circulated in a compact disc format to those who specifically requested it. As access to the Internet has grown, datasheets have been made available by direct download from the Cleveland Bay Horse Society website, backed up by continued availability of paper copies for those breeders who do not have access to electronic methods.

In addition to attracting substantial uptake in Cleveland Bay breeders through information and education regarding the aims and objectives of the breed management scheme, it was decided to support this with incentive schemes and funding from the Horse Race Betting Levy Board (HBLB), which was used to reward breeders adopting the SPARKS‐based objectives and avoiding highly inbred matings. Funding from the HBLB was also used within the UK to assist with travel grants or with the additional cost for the use of artificial insemination using fresh or frozen semen as opposed to live cover, in order to deal with constraints regarding geographical distance.

Outside of the UK, although funding restrictions prevented using HBLB monies, incentives relating to free registrations and foal grants provided from Breed Society funds were made available to offset costs of using appropriate stallions where distances involved could be considerable. In North America and Australasia, because of geographical distance, the use of artificial insemination as opposed to live cover is far more prevalent than in the UK and has helped address this issue.

As mean kinship changes with the population, with new foals entering the population, animals dying or being gelded and no longer being able to contribute to future generations, fresh datasheets were produced in January/February each year at the start of the UK breeding season, by inputting the previous year's registrations and losses into the database and recalculating the mean kinship matrix.

### 2.3 Monitoring

Although the objectives of the breed management scheme were clear—to reduce the rate of increase in inbreeding and retain as much genetic diversity as possible, it was important to establish an appropriate method of monitoring progress in achieving those targets. One of the most commonly used but also most abused and misunderstood metrics in conservation biology is effective population size (*N*_e_) which is the number of breeding individuals in an idealized population that would show the same amount of random genetic drift or the same amount of inbreeding as the population under consideration (Oldenbroek, 2007).

In reality, livestock populations are not maintained and bred as idealized populations, and many models for *N*_e_ have evolved to account for real‐world divergence from the idealized model. Census effective population size (Wright, 1931) is derived from the number of breeding males and females used as parent animals, to take account of the imbalance in the number of sires and dams being used. This model still assumes random mating which is not the case in the breeding of domestic livestock, and census effective population size usually leads to a gross overestimation of the *N*_e_ parameter. In fact, *N*_e_ is inversely related to the rate of increase in inbreeding in a population and may be derived from the classic equation *N*_e_ = 1/2 Δ_IBD_, where IBD is inbreeding determined by alleles that are identical by descent.

One well‐respected conservation biologist has been as bold as to write “Beware, do not accept any other definition for *N*_e_ other than *N*_e_ = [2Δ*F*]^−1^, even if stated in a scientific publication or textbook—they are wrong and potentially very misleading!” (Woolliams, 2007). The FAO quite rightly define *N*_e_ in terms of rate of increase in inbreeding (FAO I, 2004), and several updated models taking into account time interval and coancestry have been proposed in recent years (Leroy et al., 2013).

For this scheme, annual registrations were initially recorded in the SPARKS database and exported for analysis with ENDOG 4.8 (Gutierrez & Goyache, 2005) and more recently the web servers at PopRep (Groeneveld et al., 2009). The resulting PopRep monitoring report details a rationale cascade for selecting from six alternative methods of calculating effective population size (Groeneveld et al., 2009; Gutierrez et al., 2009).

These methods are presented in Table 2.

**TABLE 2:** Methods for estimating effective population size from PopRep (Leroy et al., 2013)

Method	Source	Formula	Description
*N*_e_‐Cens	Wright (1923)	$N_{e}=4\times \frac{S_{n}\times D_{n}}{S_{n}+D_{n}}\times 0.7$	*S_n_* = number of sires per generation *D_n_* = number of dams per generation
*N*_e_‐Δ*F*_p_	Falconer and MacKay (1996)	$\Delta F_{p}=\frac{F_{t}‐F_{t‐1}}{1‐F_{t‐1}}$	*F_t_* = ⊘ inbreeding coefficient of offspring *F_t_* _− 1_ = ⊘ inbreeding coefficient of direct parents
*N*_e_‐Δ*F*_g_	Falconer and MacKay (1996)	$\Delta F_{g}=\frac{F_{t}‐F_{t‐1}}{1‐F_{t‐1}}$	*F_t_* _− 1_ = ⊘ inbreeding coefficient of the ⊘ parents generation
*N*_e_‐Coan	Falconer and MacKay (1996)	$\Delta f_{g}=\frac{f_{t}‐f_{t‐1}}{1‐f_{t‐1}}$	*f_t_* = ⊘ additive genetic relationship (AGR) of offspring *f_t_* _− 1_ = ⊘ AGR of parents
*N*_e_‐Ln	Pérez‐Enciso (1995)	$\Delta F_{\ln}=\left(‐1\right)bL$	*b* = slope from the logarithmic regression of In(1 − *F*) on year of birth *L* = generation interval
*N*_e_‐Ecg	Gutierrez et al. (2009)	$\Delta F_{i}=1‐\sqrt[{{\text{Ecg}}_{i}‐1}]{1‐F_{i}}$	Ecg = sum of all known ancestors with ${\left(\frac{1}{2}\right)}^{n}$ *F_i_* = individual inbreeding coefficient

These methods use time windows of different lengths, and for our purposes, the method with the shortest time window will be most appropriate. The PopRep decision‐making cascade tests for both population stratification and history, as well as the stability of the data, to decide among five of the methods as to which is most suitable, leaving *N*_e_‐Cens(us) to last. The cascade is set out in Table 3. Where all conditions are met, *N*_e_‐Ln will be the preferred method. If this is not the case, selection moves down the table, with *N*_e_‐Cens being the ultimate approach.

**TABLE 3:** PopRep cascade for determining effective population size

*N*_e_‐Ln	Animals born in generation *t*
*N*_e_‐Δ*F*_p_	Animals and their parents born in generation *t*
*N*_e_‐Δ*F*_g_	Animals born in generation *t* and *t − *1
*N*_e_‐Coan	Animals born in generation *t* + 1 and *t*
*N*_e_‐Ecg	Animals with their complete ancestors born in generation *t*
*N*_e_‐Cens	Parents of animals born in generation *t*

## 3 RESULTS

### 3.1 Number of foals registered

**FIGURE 4:**
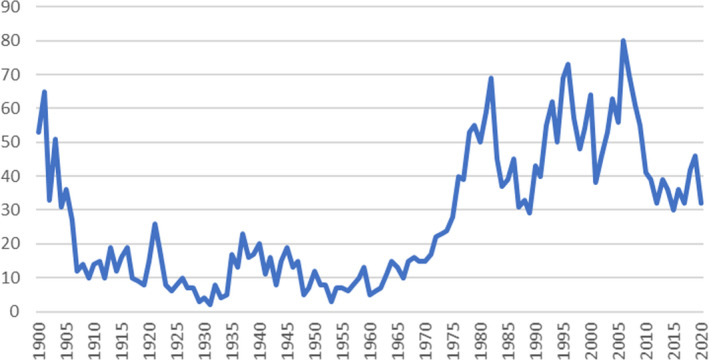
Number of foals registered per year 1900–2020 in the Cleveland Bay horse breed

Although the uptake of such a scheme raised societal and public acceptance queries, due to the education and support campaign administered simultaneously to the development of the framework for breed management, there has been an increase in foal registrations since 1960; since the breed management program was established, foal registrations have been relatively maintained across the last 16 years (Figure 4).

### 3.2 Scheme compliance

**FIGURE 5:**
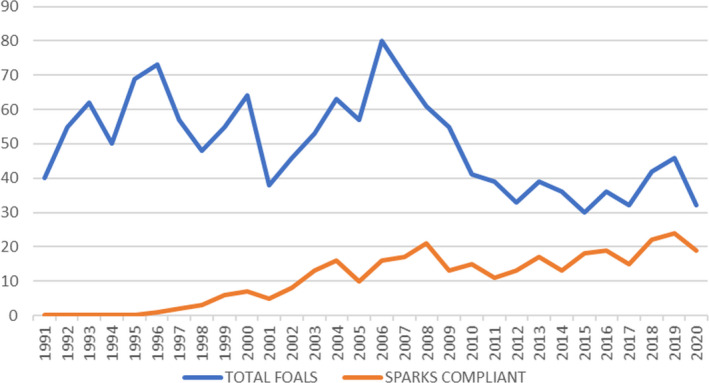
Compliance of registrations to SPARKS criteria 1990–2020

As participation in the scheme was on a voluntary basis, it was impossible to expect full uptake. In order to evaluate any possible beneficial effects, it was important to consider the level of compliance by breeders and to determine whether there had been any change in breeding practice. This is illustrated in Figure 5.

**FIGURE 6:**
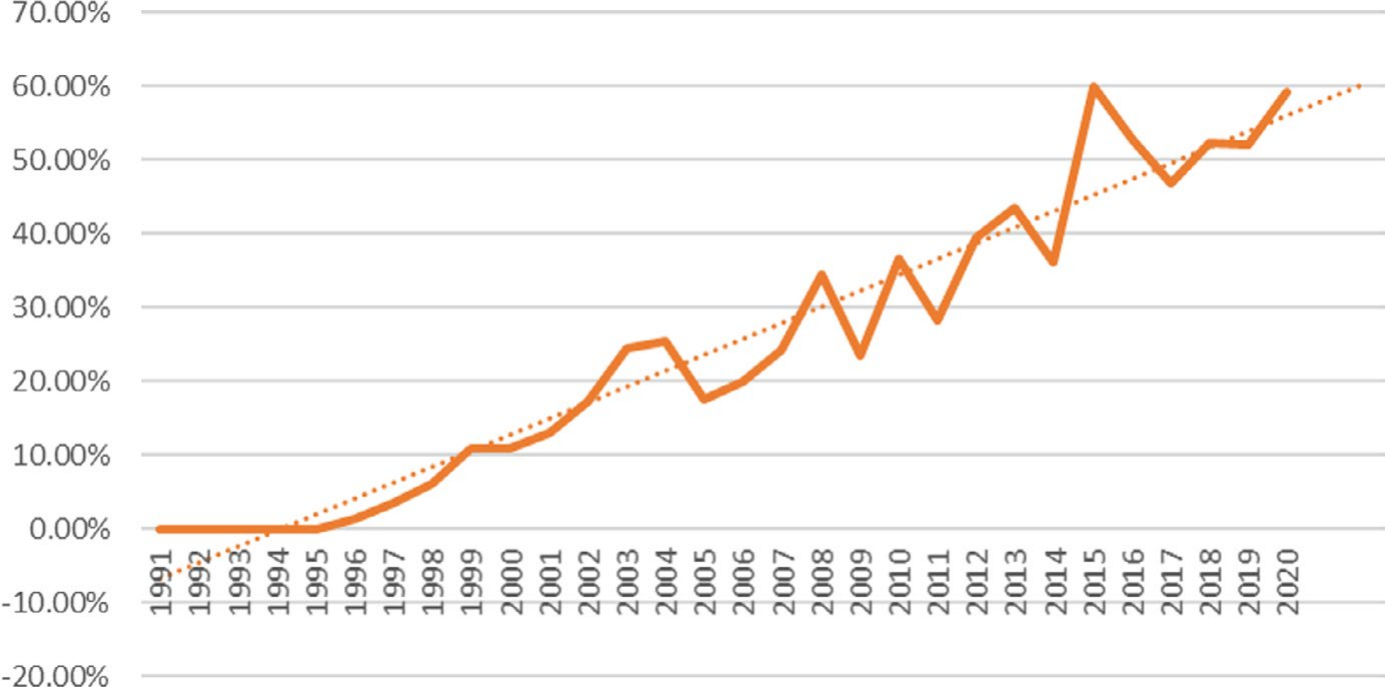
Percentage of registrations complying with SPARKS criteria 1990–2020

As the level of compliance appears to have shown a steady increase, the level of registrations has fluctuated, with a notable decline following the financial crisis in 2008. In order to determine a more accurate assessment of scheme uptake, the level of compliance as a percentage of registrations is illustrated in Figure 6.

Prior to 1999, the level of compliance of registrations to the guideline criteria was under 10%. In that year, breeders becoming aware of the availability of data made informal requests for coancestry tables with which to guide mating decisions. These were issued on a private basis to breeders in the UK and overseas. There was a particularly heavy demand for data from breeders in North America. On average, approximately 20 sets of figures were issued per annum over the period 1999–2004, before the breed management scheme was officially adopted by the CBHS, and datasheets were universally distributed to mare owners. After 1999, the number of registrations that complied with the criteria grew steadily to reach over 25% by 2004 when the scheme was officially launched. In the subsequent 16 years, although annual compliance figures have fluctuated, the trend has seen increases such that by 2020, 60% of all registrations were SPARKS compliant, that is, parent animals were of similar mean kinship with coancestry coefficients of progeny lower than the average inbreeding coefficient.

### 3.3 Changes in inbreeding

**FIGURE 7:**
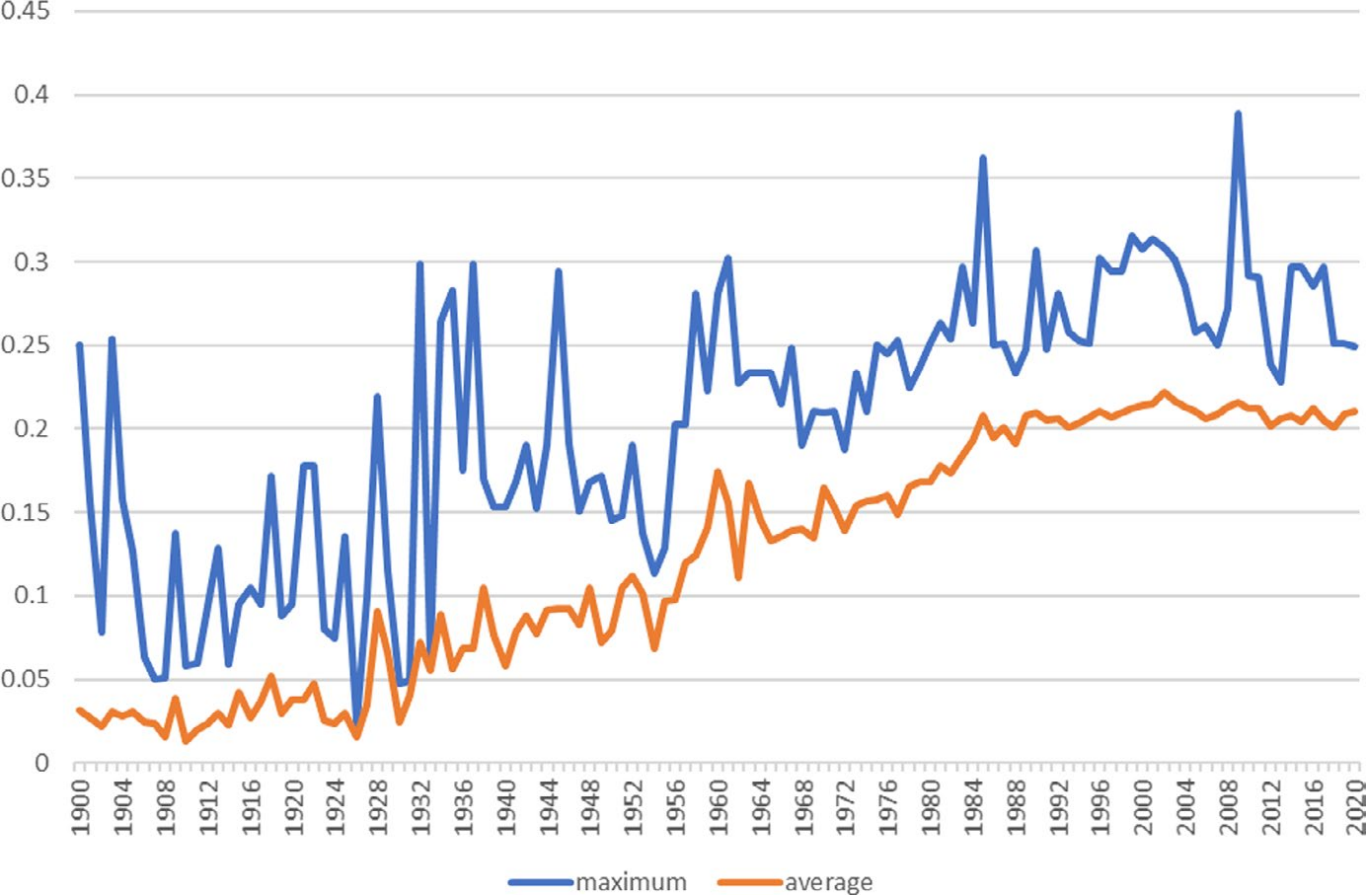
Maximum and average inbreeding 1900–2020 Source: PopRep (data in Appendix S1)

Figure 7 shows the maximum and average inbreeding within the Cleveland Bay horse breed over the past 120 years. The trend seen an increase throughout the 20th century rising to over 22% by the year 2000. Since 2004, the rate of increase has slowed considerably, concurrent with the implementation of the breed management scheme.

**FIGURE 8:**
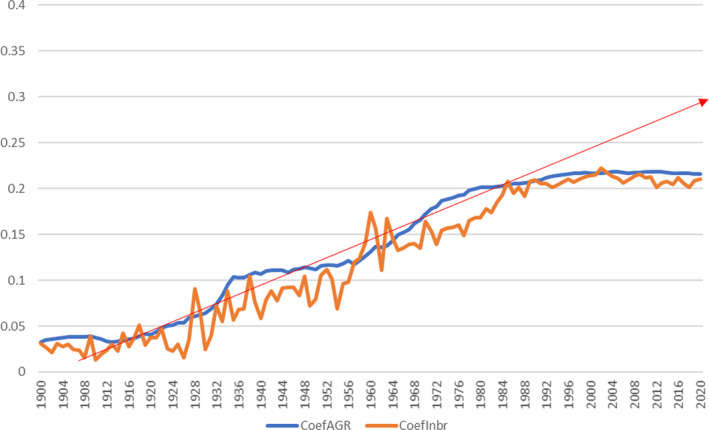
Additive genetic relationship (AGR) and average inbreeding coefficient 1900–2020 Source: PopRep (data in Appendix S1)

Figure 8 illustrates the near‐linear increase in additive genetic relationship and average inbreeding coefficient in the Cleveland Bay horse population between 1900 and 1995. With an average generation interval of 9 years, this period of time represents over 10 generations. By 1990, both additive genetic relationship and average inbreeding coefficient of the global Cleveland Bay horse population had exceeded 0.20.

**FIGURE 9:**
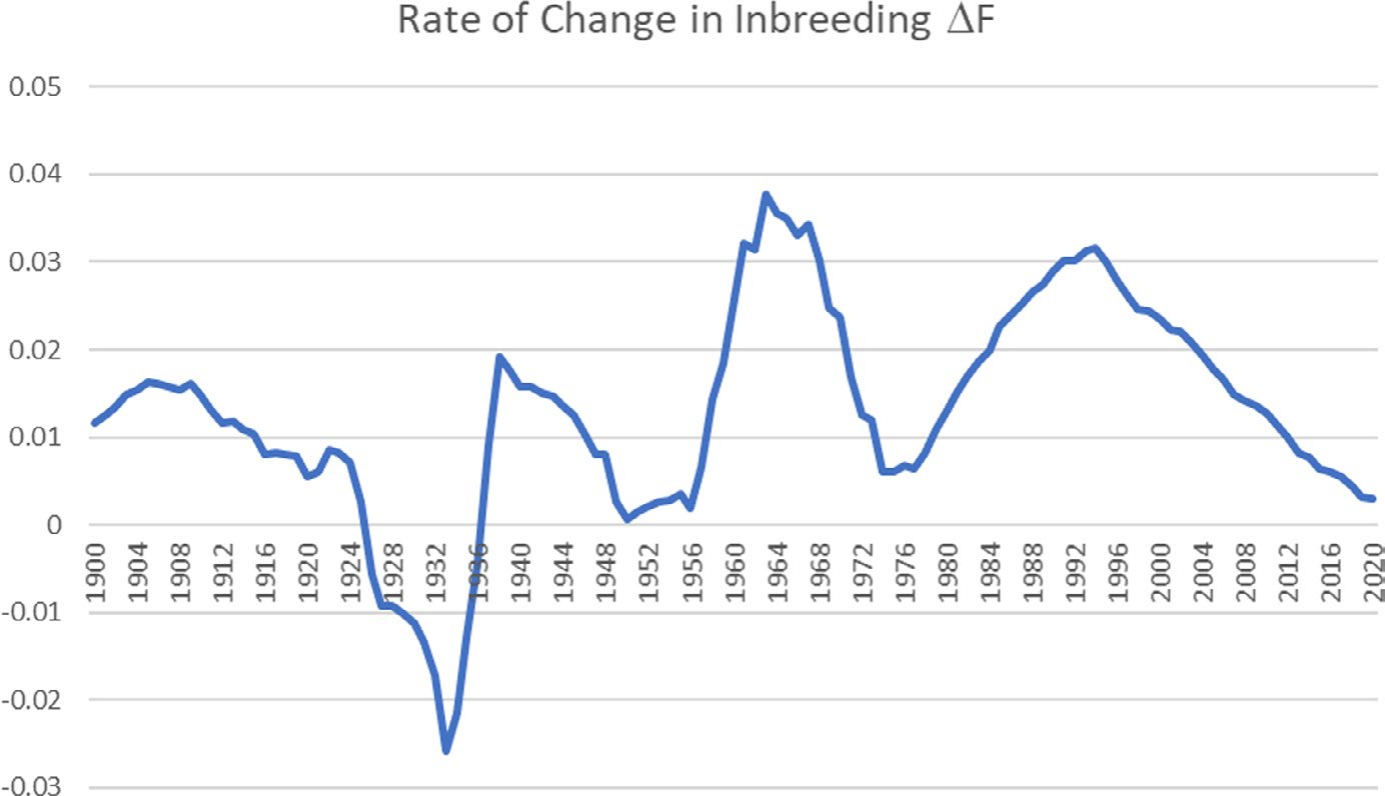
Rate of increase in inbreeding in the Cleveland Bay horse from 1900 to 2020

The pattern of change in Δ*F* over the period 1900–2020 is shown in Figure 9. The period up to 1980 shows some with the rate varying widely about a mean of 0.01. The widely ranging figures are best explained by the relatively small population size and the disproportionate year‐to‐year influence of a few highly inbred progeny, including putting delta F into negative figures. Between 1980 and 1994, the pattern of decrease seen since 1963 ends, with a peak of 0.03 in 1993. Since 1995, the trend has been for a decrease, possibly concurrent with the increased awareness of the need to manage inbreeding brought about by the Walling report and with the implementation of the SPARKS breed management scheme and freely available advice to breeders aimed at controlling the rate of increase in inbreeding.

After 1995, the rate decreases reaching a minimum of 0.0029 in 2020. This coincides with the period during which the breed management program was actively promoted by the CBHS.

### 3.4 Effective population size

The ultimate objective of the breed management scheme was to bring about an increase in *N*_e_, by controlling the rate of increase in inbreeding. The results of all six methods for calculating effective population size are set out in Table 4. Using the decision‐making cascade set out in Table 3, the recommended method is using *N*_e_‐Δ*F*_p_, which is calculated to be 171 at the end of the 2020 registration year.

**TABLE 4:** Evaluation of *N*_e_ by six methods using PopRep (Groeneveld et al., 2009)

*N*_e_‐method	2020	2019	2018	2017	2016	2015	Data history
*N*_e_‐Cens	175	180	187	200	207	204	2009–1999
*N*_e_‐Δ*F*_p_	171	160	114	91	83	77	2020–1999
*N*_e_‐Δ*F*_g_	−72	−96	−135	−168	−138	−168	2020–1999
*N*_e_‐Coan	−183	−352	−685	−568	−219	−397	2031–2010
*N*_e_‐Ln	−59	−45	−47	−160	−226	−79	2020–2010
*N*_e_‐Ecg	31	31	30	30	30	30	2020–1723

Note

Proposed *N*_e_: *N*_e_‐Δ*F*_g_ = 171.

**FIGURE 10:**
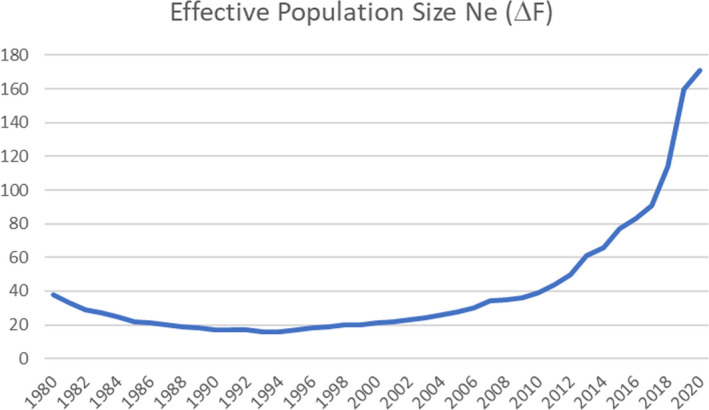
Effective population size from rate of change of inbreeding 1980–2020

Figure 10 shows the change in *N*_e_ over the 40‐year period 1980–2020. The prime reason for controlling the rate of increase in inbreeding is to bring about an increase in *N*_e_. Between 1993 and 2020, there is a significant increase in *N*_e_, from 19 to 171. Since 2012, *N*_e_ has remained above the FAO's recommended minimum of 50 for a viable population.

### 3.5 Highly inbred matings

Figure 7 shows the pattern of average and maximum inbreeding in the Cleveland Bay population. Over the years, the peak levels of inbreeding have varied substantially under random mating.

Table 5 sets out the occurrence of highly inbred matings since 1983.

**TABLE 5:** Number of highly inbred matings per year

Year	>0.24	>0.25	>0.26	>0.27	>0.28	>0.29	>0.30	>0.31	>0.32	>0.33	>0.34	>0.35	>0.36	>0.37	>0.38	>0.39	>0.40
1983							1										
1984		1	1														
1985	3		2			2				1			1				
1986	1		2														
1987	2	1	1														
1988	2																
1989	3	2															
1990		2			3												
1991	1	2															
1992	3	3	2		1												
1993	5	1	3														
1994	4	1	1														
1995	6	4	1														
1996	6	3	1	1	1												
1997	8	3	1	1													
1998	2	4		1			1										
1999	4	1	1			1			2								
2000	3	7		1				2									
2001	3	3	1						1								
2002	2	4	1			1	1	1									
2003	5	1	1	1				1									
2004	3	3	1			1											
2005	2	2	2														
2006	2	5	1	1													
2007	1	5															
2008	2	4	2	1	1												
2009	1	2						1								1	
2010	1				1	2											
2011	1	1	2			1											
2012	1																
2013																	
2014		2					1										
2015							1										
2016	1	1	2		1	1											
2017	2	1					1										
2018	1		1														
2019	2		1														
2020	1	1															

The data are presented graphically in Table 5, which shows the reduction in occurrence of highly inbred matings (>0.25 to >0.39) since 2009. A level of inbreeding of 0.25 is equivalent to full‐sib mating. It would seem that the advisory scheme has helped reduce the number of potentially genetically damaging highly inbred matings that take place. By making the data widely available, although breeders may not—by choice or due to geographic limitations—be able to fully comply with the scheme, they may be more easily able to avoid the mating of closely related individuals.

## 4 DISCUSSION

The Cleveland Bay horse is one of seven equine breeds listed as priority breeds by the Rare Breeds Survival Trust in their 2021–22 Watchlist. Over the past 100 years, the number of annual registrations has fluctuated significantly under social, economic, and environmental pressures. The number of registrations was at a minimum of 2 in 1931, at a time of great economic depression in the interwar years. It reached a maximum of 80 registrations in 2006, immediately before the global financial crash in 2008, after which numbers registered each year have reduced substantially. In the years following World War II, the Cleveland Bay Horse population went through a demographic bottleneck (Dell et al., 2020b) when the UK stallion population was reduced to just 4 animals and the number of foal registrations reduced to only three in 1953.

The closed nature of the studbook and the limited number of founder animals have both contributed to an increase in inbreeding over time, such that by the year 2000, average inbreeding was at 21.39%. The restricted population size has had substantial influence on the fluctuating rate of change in inbreeding, throwing it into negative figures in the period 1925–1935. In the postwar years 1950–1960, the population, in recovery from demographic bottleneck, saw a rapid rise in the rate of increase in inbreeding, before falling; during the subsequent 15 years (Ballou & Lacy, 1995), the rate of increase in inbreeding was increasing too such that by 1995, the effective population size measured from the rate of increase in inbreeding had decreased to less than 20.

It is widely accepted that populations with an effective population size below are unviable (Dell et al., 2020b; Frankham et al., 2002), and digitization of the Cleveland Bay studbook in the mid‐1990s made it an ideal candidate for a breed management program aimed at maximizing retention of genetic diversity through minimizing the rate of increase in inbreeding. In 2004, there was no evidence that adopting best practice, developed in the conservation of captive wildlife populations (Ballou & Lacy, 1995), and adapting that to the world of livestock breeding would be effective in stemming the rate of increase in inbreeding and reduction in effective population size, both of which were clear indicators of the unsustainable loss of genetic diversity that was taking place in the Cleveland Bay breed and a significant threat to its survival.

Since it was first introduced as a practical tool for breeders to aid choosing mating pairs of similar mean kinship, producing progeny of the lower‐than‐average inbreeding coefficient, the SPARKS breed advisory scheme has contributed to a noticeable reduction in both the rate of increase in inbreeding and additive genetic relationship, as illustrated in Figure 8. The trend line shows that had AGR and inbreeding remained unchecked by 2020, they may well have both reached 30%, with 25% inbreeding being equivalent to the mating of full siblings or of parent animals with their own progeny. However, the results of the PopRep analysis show that since 2004, the AGR has remained virtually constant at 21%, whereas average inbreeding has fluctuated between 20% and 21%.

As a consequence of minimizing the rate of increase in inbreeding, effective population size has shown a substantial improvement. From a minimum of 16 in 1996, the year in which advances in computer software led to the ability to calculate coancestry coefficients for every potential male–female cross, effective population size has increased such that by 2012, it was at 50, the size above which the FAO of the United Nations recommends that livestock populations should be maintained (FAO I, 2004). Between 2012 and 2020, effective population size has continued to increase, passing 100 in 2018 and reaching 171 in 2020.

Although there will always have been a background of matings that would result in progeny of a lower‐than‐average inbreeding coefficient prior to the introduction of the SPARKS breed management scheme, this level was at approximately 20% in 2004 at its introduction. Since that year, compliance has increase in an approximately linear manner as an increasing number of breeders have become aware of and adopted the advice and acknowledge the benefits to the breed as a whole. Moreover, the combination of the SPARKS‐based theory with that of the population reports, similar to those obtained from PopRep, have led to the establishment of the BCAS. This suite of programs allows for breed management reports to be produced, as well as obtaining population genetic information linked to these in order to assess the effects of the management schemes on the population genetic parameters each year, all within a single computational framework.

Compliance peaked at 60% in 2015 and again in 2020, no doubt assisted by financial incentive to breeders to breed compliant foals, provided by targeted allocation of annual grant money from the Horserace Betting Levy Board. This has brought significant improvements in effective population size and demonstrates that it is possible to manage the rate of inbreeding in a globally dispersed rare breed where individual breeders are ultimately responsible for mating choices.

The EU Zootechnical Regulations 2016 (post‐Brexit the UK Animal Breeding Regulations 2019) state that breeding selection must remain a choice made by breeders and that it cannot be prescribed by any breed society or other authority. By making breed management advice freely available to breeders in a format from which they can easily identify potential matings that will benefit the breed as a whole while still enabling them to exert freedom of choice on the stallion phenotype, the Cleveland Bay Horse Society has achieved that aims of the breed management scheme while complying with the current legislation.

What has become increasingly clear throughout this project is that management of rare equine breeds is as much about winning the “hearts and minds” of breeders as it is about managing the animals themselves. Prior to the introduction of the scheme, there appears to have been a “background compliance” of about 20%, being a reflection on the “random” uncoordinated efforts of the dedicated core of Cleveland Bay breeders. It is probable that this “hard core” of breeders did not change their practice as a result of the scheme. The increase in compliance probably came about by encouraging newer breeders and tapping into fresh enthusiasm for conservation of a rare breed.

Although some breeders have accepted the need to control the rate of inbreeding and adopted the scheme as part of their “toolbox” for stallion choice, others have found it too restricting. In part, this is because of geographical restrictions, with no compatible stallion within easy traveling distance. This difficulty has been less apparent in North America, where the scheme has been accepted by the vast majority of breeders. This may in part be due to the much greater use of artificial insemination (AI) techniques for breeding than the UK, where live cover is still by far the norm.

It has also been apparent that a continuing program of education for breeders about the benefits of and need for any sort of breed management scheme is essential. The emphasis of that program has to be stressing that such a scheme can only ever be voluntary and does not detract from individual breeder choice, where selection can be made based on the phenotype as well as genotype.

One benefit of the scheme that has contributed to its success has been the reduction in the number of highly inbred matings taking place each year (those producing progeny with inbreeding coefficient greater than 0.24). For breeders who for whatever reason are not able to undertake the most advantageous “SPARKS compliant” matings, there appears to be a recognition that highly inbred matings are damaging to the future of the population, contributing to the expression of deleterious alleles and increasing homozygosity. The data illustrated in Table 5 reveal that since 2010, there has been a reduction in the frequency and inbreeding coefficient of these highly inbred matings, both of which will have contributed to the increasing effective population size.

The increasing use of the Internet has proven to be both an advantage and a distraction as the scheme had developed. Although it can provide a means through which breed societies can disseminate information and educate breeders, there is also an expectation from an increasing number of breeders for its use to provide open access to data. Since 2010, the Cleveland Bay Hose Society of North America has provided access to the whole set of SPARKS breeders' datasheets on its website, and the Cleveland Bay Horse Society in the UK followed suit in 2014.

As more breeders accept the data in the present mare‐based form, there has been a growing call for it to be available in stallion‐based form too. In the main, this has been from colt owners who want to seek out female animals to purchase, which would be suitable for their own “in‐house” breeding programs, and not from stallion owners wanting to use the information for commercial gain. These data have been made available to stallion owners from 2020. There is concern that this could lead to any one year's crop of foals being sired by a limited number of sires and compromising effective population size when measured by census methods.

Over the 16 years of the scheme, there have been substantial developments in computer processors and computing software. The original SPARKS software was a 16‐bit program, and GENES was a dos routine. To use the software on a modern 64‐bit machine running Windows 10 requires the use of emulation software such as DosBox. Indeed, as datasets become larger, it becomes necessary to adopt more appropriate methods to calculate the kinship matrix, which otherwise becomes time‐consuming routines requiring considerable processing power. However, the open‐source statistical environment R provides a solution to this issue, and the package OPTISEL (Wellmann, 2019) can rapidly calculate the required metrics.

The ability to integrate most common database formats with R statistics through an API (application programing interface) provides a route for the next generation of cloud‐based breed management advisory schemes to succeed the one described in this article.

Developments in breed management theory and software continue, and at present, optimization of genetic contributions continues to be the preferred method for managing the majority of livestock breeds (Meuwissen, 2009). However, it is recognized that in very small populations, the priority is to breed from as many males and females as possible and that selection based on estimated breeding values based on phenotypic traits is inappropriate (Oldenrboek et al., 2014). Although stallion selection based on optimum genetic contributions might be effective in breeds such as the Jutland horse (Nielsen & Kargo, 2020), it remains the case that for our most endangered equine population, control of increase in inbreeding through management of mean kinship remains the most appropriate method.

The development of gene sequencing techniques using SNPs provides opportunity to assess levels of inbreeding through molecular methods (ROH), and such studies are now being reported for a number of equine breeds (McGivney et al., 2020; Todd et al., 2018). It has been suggested that this new technology will replace the use of pedigrees in formulating breeding plans (McGivney et al., 2020). However, it has also been established that where a pedigree is robust and deep, it can provide information that is consistent with that confirmed by analysis of ROH (runs of homozygosity) (Todd et al., 2018) and breeds maintaining robust studbooks spanning the centuries such as the Thoroughbred and many traditional sport horses as well as native equine breeds will find analysis using pedigree‐based methods a cost‐effective alternative.

## 5 CONCLUSIONS

At the time of writing, the Cleveland Bay SPARKS breed management scheme has been in place for an excess of sixteen years. With an average generation interval of 9 years, this represents almost two complete generations. No other such breed management program for equines of this length has been reported in the literature. Although the results reported here are encouraging, it must be remembered that for the scheme to have maximum benefit, it would have to run for five generations or 45–50 years. It is hoped that over that period, inbreeding will increase at less than 1% per generation, as recommended by the FAO (FAO I, 2004) and that through bringing together all of these computational tools within our BCAS framework, we are able to support breed management on such a scale much more widely.

The scheme has been demonstrated to bring a significant improvement in effective population size in a globally endangered equine population that is controlled not by a single population manager but by individual breeders operating with advice from an umbrella breed society. In the more controlled environment of captive wildlife conservation, management of mean kinship continues to be the population management method of choice. The success of management of captive wildlife populations has been well‐tested (Margan et al., 1998; Montgomery et al., 1997) and has recently been reported in conservation of species such as Mexican and red wolves (Hedrick & Fredrickson, 2008), lowland tapirs (Goncalves da Silva et al., 2010), and European bison (Daleszczyk & Bunevich, 2009). In commercially viable livestock, species management through BLUP or optimum genetic contributions is now commonplace (Banks & Brown, 2009; Colleau & Moureaux, 2006; Meuwissen, 2009; Mrode et al., 2009; Pong‐Wong & Woolliams, 2007). Prior to this study, the closest comparable project has involved icelandic sheepdogs (Oliehoek et al., 2009) which has extensively reviewed and investigated the potential for maintaining diversity through controlling mean kinship. The present study provides evidence of the benefits of the practical implementation of breed management using the same methods. Schemes of a similar nature will be of benefit to other endangered breeds as well as other types of livestock.

Since 2017, SPARKS‐type breed management advice has been made available via the RBST to two more of the seven priority equine breeds on the watchlist, these being the Eriskay Pony Society and the Suffolk Horse Society. In addition, similar data have been used to help manage the RBSTs' own herds of critically endangered Vaynol cattle.

In 2019, SPARKS data were used to select the most appropriate Suffolk stallion to be used from sexed semen trials and to inseminate a Suffolk mare belonging to Nottingham Trent University. The successful birth of a filly foal has been widely reported in the UK National Press, and a report on that project is in preparation.

## CONFLICT OF INTEREST

None declared.

## AUTHOR CONTRIBUTIONS

**Andrew Dell:** Conceptualization (equal); Data curation (equal); Formal analysis (equal); Investigation (equal); Methodology (equal); Resources (equal); Validation (equal); Visualization (equal); Writing‐original draft (equal). **Mark Curry:** Conceptualization (equal); Investigation (supporting); Methodology (supporting). **Elena Hunter:** Formal analysis; Project administration (equal); Validation; Visualization; Writing‐review & editing (equal). **Ruth Dalton:** Data curation (equal); Formal analysis (equal); Software (equal). **Kelly Yarnell:** Data curation; Formal analysis; Project administration (equal); Resources; Supervision (equal); Validation; Writing‐review & editing (equal). **Gareth Starbuck:** Data curation; Formal analysis; Funding acquisition (equal); Project administration; Resources; Supervision; Validation; Writing‐review & editing. **Philippe B. Wilson:** Conceptualization (equal); Data curation (equal); Formal analysis (lead); Funding acquisition (equal); Project administration (equal); Resources (equal); Software (equal); Supervision (equal); Validation (equal); Visualization (equal); Writing‐original draft (equal); Writing‐review & editing (equal).

## Supporting information

Appendix S1

## Data Availability

Data are archived in our open‐access Figshare repository located at https://doi.org/10.6084/m9.figshare.16437339 and included SPARKS datasheets, population reports, and base datasets.

## Footnotes

* The use of datasheets since 2004 has highlighted the subtle difference between Mean Kinship of parents and Kinship Coefficient of progeny. Until new animals are registered and entered onto the system we cannot calculate their Mean Kinship or Kinship Band. This highlights the importance of prompt registration of pure‐bred foals. Owners are advised to follow the above guidelines in making breeding choices, as they continue to be the most robust and scientifically validated method of ensuring the genetic wellbeing of the global Cleveland Bay population.
