# Supplementary material for: 16 Years of breed management brings substantial improvement in population genetics of the endangered Cleveland Bay Horse
Source: Ecol Evol. 2021 Oct 3;11(21):14555–72. doi: 10.1002/ece3.8118 (PMC8571631; doi:10.1002/ece3.8118)
Supplement: Supplementary file 1 — Appendix S1 [file ECE3-11-14555-s001.pdf]

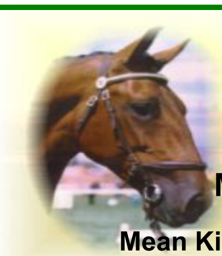

Cleveland Bay Horse Society

SPARKS Kinship Table 2021

For Pure-bred Mares / Stallions Licensed For Pure Bred Breeding

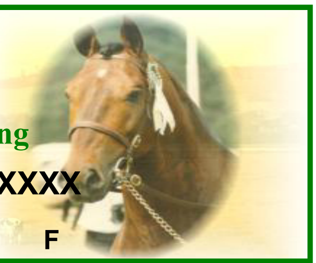

Mare's Name <u>XXXXXXX</u> **Stud Book Number XXXX**

Mean Kinship 0.2348 Inbreeding Coefficient .2264 Mean Kinship Band F

NB THIS SHEET AND THE DATA IT CONTAINS IS ONLY VALID FOR THE 2021 BREEDING SEASON

Please follow the traffic lights & use in conjunction with the accompanying guidance notes

Tier 1 Matings highlighted in GREEN are SPARKS compliant & are ENCOURAGED

Tier 2 Matings highlighted in YELLOW are not fully compliant but are the "BEST OF THE REST"

Tier 3 Matings highlighted in ORANGE are not compliant AND jump Bands which is DISCOURAGED

Tier 4 Matings highlighted in RED should be AVOIDED as they are highly inbred (> 0.24)

Stallion	Studbook Number	Stallion's Inbreeding Coefficient	Stallion's Mean Kinship	Kinship Band	Inbreeding Coefficient of Progeny	Availability	Stallion's Location
Cholderton Cacus	M2495	.156	0.1993	B	.1918	CONTACT OWNER	ENGLAND
Cholderton Ixion	M2661	.1714	0.2063	C	.2006	Live Cover, Chilled & Frozen	UK
Wyevale Brayden	M2665	.1988	0.2149	D	.2005	Live Cover & Frozen UK	ENGLAND
Penrhyn Romulus	M2615	.2298	0.2136	D	.2009	Contact Owner	WALES
Cholderton Icarus	M2662	.175	0.2159	D	.2037	Live Cover Only	UK
Cholderton Jupiter	M2727	.1832	0.2100	D	.2101	Live Cover Only	UK
Folds Victor	M2767	.1908	0.2173	D	.2234	Gelded (Frozen Only)	UK
Fenton	M2531	.2032	0.2196	D	.2238	Live Cover Only	WALES
Penrhyn Sextus	M2584	.2431	0.2221	E	.2078	Contact Owner	WALES
Brackenbrae Somerled	M2842	.222	0.2247	E	.2136	Live Cover Only	UK
Brackenbrae Stormcloud	M2853	.222	0.2245	E	.2136	Live Cover & Chilled	UK
Kirkmoor Wolfsbane Flower	M2782	.2088	0.2256	E	.2196	Live Cover Only	ENGLAND
Highpasture Fellowship	M2589	.2189	0.2206	E	.2205	Live Cover & Frozen UK	WALES
Wyevale Wot A Charmer	M2596	.2199	0.2285	E	.2215	Live Cover & Chilled	UK
Folds Director	M2846	.1946	0.2228	E	.2216	Live Cover;Chilled & Frozen	ENGLAND
Carolina Charlie Boy	M2718	.2591	0.2288	E	.2224	Live Cover Only	UK
Pembridge Justice	M2787	.2353	0.2252	E	.2247	Live Cover Only	ENGLAND
Wyevale Boomerang	M2688	.207	0.2260	E	.2272	Contact Owner	UK
Rosemeadow Springtime	M2770	.2288	0.2263	E	.2274	Live Cover Only	ENGLAND
Botton Grove Brandon	M2668	.2172	0.2293	E	.2321	Live Cover Only	ENGLAND
Jemoon Highwayman	M2824	.232	0.2293	E	.2325	Contact Owner	UK
Earlswood Traveller	M2649	.2193	0.2290	E	.2341	Live Cover Only	ENGLAND
Willow Troy	M2577	.2058	0.2279	E	.2386	Live Cover Only	ENGLAND
Penrhyn P.S	M2764	.2122	0.2302	F	.2183	Live Cover UK /Frozen AUS	ENGLAND
Beamish Fugleman	M2540	.1996	0.2312	F	.2249	Live Cover Only	ENGLAND
Stainmore Scotch On The Rocks	M2830	.1996	0.2305	F	.2249	Live Cover & Frozen UK	ENGLAND
Thomsonbrook King William	M2737	.2102	0.2322	F	.2306	Live Cover Only	UK
Blackclough Kilkenny	M2582	.2208	0.2311	F	.2307	Live Cover Only	UK
Stainmore Lochinvar	M2799	.226	0.2326	F	.2308	Live Cover Only	ENGLAND
Barbarian Gigalo	M2795	.2266	0.2310	F	.2321	Contact Owner	ENGLAND
Billy's Joint Account	M2822	.2314	0.2350	F	.2329	Contact Owner	UK
Crosswaysfarm Albion Bomber	M2882	.2215	0.2306	F	.2339	RBST GENEBANK	ENGLAND
Mulgrave Royal Emperor	M2873	.3057	0.2329	F	.2345	Contact Owner	UK
Braithwaite Challenger	M2848	.221	0.2330	F	.2351	Live Cover & Chilled	UK
Lindon Principal	M2558	.2283	0.2351	F	.2362	CONTACT OWNER	ENGLAND
Hallhouse Gladimere	M2836	.2253	0.2314	F	.2372	Live Cover & Chilled	UK
Tynedale St Osmund	M2862	.225	0.2340	F	.2375	Contact Owner	ENGLAND
Kirkmoor Elderberry Flower	M2636	.211	0.2361	F	.2381	Live Cover & Frozen UK	UK
Barbarian Hustler	M2834	.2251	0.2335	F	.2383	Contact Owner	UK
Beamish Touch'N'Go	M2549	.2399	0.2378	F	.2417	Live Cover Only	ENGLAND
High Park Schoffield	M2487	.2174	0.2378	F	.2468	UK EU USA AUS NZ	ENGLAND
Holmside Applejack	M2736	.2321	0.2397	F	.2619	Live Cover Only	ENGLAND
Belladonna Isaac	M154US	.2298	0.2136	D	.2009	Contact Owner	CANADA
Gaylord Edward Of York	M261US	.1957	0.2147	D	.2128	Contact Owner	USA
Cholderton Issus	M2676	.2038	0.2170	D	.2177	Live Cover Only	IRELAND
Idlehour Yorktown	M103US	.1908	0.2157	D	.2179	CONTACT OWNER	USA
Windy Day Shakespeare	M235US	.2998	0.2223	E	.2086	Contact Owner	USA
Frostyoak Hampton	M256US	.1979	0.2221	E	.2213	Contact Owner	USA

Idlehour Lion's Share	M140US	.2008	0.2240	E	.2233	Live Cover Only	USA
Arena Maestro	M2828	.2165	0.2212	E	.2235	Live Cover & Chilled	USA
Galveston	M236US	.2146	0.2244	E	.2237	Contact Owner	USA
Pembridge Joshua	M2682	.2353	0.2252	E	.2247	FRESH/CHILLED	USA
Bayfield Bodhi	M253US	.2239	0.2294	E	.2257	Contact Owner	USA
Lord Brigadoon	M272US	.2558	0.2209	E	.2329	Contact Owner	USA
Billara Alfredo	M234AU	.286	0.2261	E	.2361	CONTACT OWNER	AUSTRALIA
Texlea Impeccable Marksman	M147AU	.2322	0.2292	E	.2364	CONTACT OWNER	AUSTRALIA
Texlea Saint Oliver	M175AU	.2714	0.2296	E	.2411	CONTACT OWNER	AUSTRALIA
Thickley Norman	M2522	.2119	0.2235	E	.2426	AI / FROZEN	CANADA
Epiphanybay Jacob	M262US	.229	0.2272	E	.2499	FROZEN ONLY	USA (Gelded)
Old Dominion Artorius	M149US	.2179	0.2254	E	.4156	Contact Owner	USA
Stainmore Wolfhound	M2521	.1996	0.2308	F	.2249	Contact Owner	USA
Peterscreek Royal Saga	M214US	.2241	0.2314	F	.2376	CONTACT OWNER	USA
Bensington Bluestone	M2627	.226	0.2379	F	.2499	CONTACT OWNER	USA
Bearpaw Waylon	M295US	.2317	0.2340	F	.2499	Contact Owner	USA
Tregoyd Topper	M2545	.2209	0.2304	F	.2523	Live Cover Only	AUSTRALIA
Tregoyd Journeyman	M2499	.2229	0.2306	F	.2549	USA Contact Owner/Frozen	USA
Epiphanybay Issac	M263US	.2267	0.2336	F	.2617	Contact Owner	USA
Old Dominion Brego	M202US	.2309	0.2328	F	.2943	CONTACT OWNER	CANADA
OldDominion Sir Galahad	M187US	.2549	0.2330	F	.3356	CONTACT OWNER	USA
Penrose Claudius	M2537	.237	0.2332	F	.3704	Live Cover Only	USA
Belladonna Pendleton	M173US	.171	0.2058	C	.2019	FROZEN ONLY	USA (Gelded)
Cholderton Boreas	M2485	.2389	0.2093	C	.2044	FROZEN UK	DECEASED UK
Southbrook Spellbound	M2345	.2084	0.2146	D	.2011	FROZEN ONLY	ENGLAND
Foxhollows (USA) Navigator	M104US	.2185	0.2159	D	.2073	FROZEN ONLY	DECEASED USA
Oakenbank Grenadier	M2326	.1722	0.2181	D	.2087	Frozen UK EU AUS NZ	DECEASED UK
Laughton Saladin	M2190	.1495	0.2155	D	.2179	Frozen UK AUS NZ	DECEASED UK
Bantry Bere	M2132	.1852	0.2213	E	.2018	Frozen UK AUS NZ US	DECEASED UK
Cholderton Whitethorn	M2366	.1897	0.2244	E	.2162	CONTACT OWNER	DECEASED UK
Fryup Marvel	M2086	.1457	0.2240	E	.2203	FROZEN US ONLY	DECEASED USA
Whitehouse Statesman	M2080	.165	0.2236	E	.2214	FROZEN UK	DECEASED UK
Forest Field Day	M2002	.2004	0.2298	E	.2308	US CAN AUS NZ(UK EU?)	DECEASED USA
Alabai Mulberry Crumpet	M197AU	.2094	0.2249	E	.2533	FROZEN ONLY	AUSTRALIA (Gelded)
LLewellyn	M244US	.2331	0.2224	E	.2618	Gelded (Frozen Only)	USA
Cholderton Yobi	M2423	.237	0.2317	F	.226	UK EU USA AUS NZ	DECEASED UK
Borderfame Prince Charming	M2251	.2103	0.2326	F	.2283	FROZEN UK AUS NZ USA	DECEASED UK
Tynedale St Dominic	M2593	.2108	0.2327	F	.2287	FROZEN US ONLY	DECEASED USA
Captain Hornblower	M2356	.2621	0.2374	F	.2335	FROZEN UK	DECEASED UK
Arena Big Ben	M2775	.2135	0.2320	F	.2337	FROZEN UK AUS NZ USA	UK
Penrhyn Dictator	M2288	.2244	0.2321	F	.2343	FROZEN ONLY	DECEASED UK
Forest Fellow	M2395	.2308	0.2341	F	.236	AI/FROZEN	CANADA
Pembridge Midshipman	M2510	.2772	0.2328	F	.2383	USA AUS NZ	DECEASED UK
Timberlane Huckleberry	M2389	.2288	0.2386	F	.2475	FROZEN UK AUS NZ USA	DECEASED UK

NB THIS SHEET AND THE DATA IT CONTAINS IS ONLY VALID FOR THE 2021 BREEDING SEASON

Please read and understand the accompanying guidance notes before using this data

NO HBLB GRANTS WILL BE PAID FOR TIER 4 (RED) HIGHLY INBRED MATINGS

CLEVELAND BAY HORSE SOCIETY - 2021

Guidance on the use of SPARKS Data Sheets

INTRODUCTION

SPARKS is a breed advisory scheme designed to promote the genetic health of the global Cleveland Bay Horse population. The data sheets have been produced annually since 2004, as an aid to Mare owners when selecting prospective stallions. They are based on pedigree & genetic analysis of the studbook using the SPARKS and GENES software packages. They do not look at the physical attributes of any stallion or potential progeny. **Mare owners must continue to use their own best judgment regarding this.**

The analysis calculates **Mean Kinships** for every known pure-bred Cleveland Bay in the global population registered in the CBHS Studbook. This is a measure of how related an individual is to every other living Cleveland Bay. It also calculates the potential inbreeding resulting from every possible male / female pairing within the population.

Each datasheet provides a **Mean Kinship figure** for a named mare and assigns the mare to a **Mean Kinship Band** (A to G) based on this figure.
The table names every licensed stallion and orders them by:

- **i** their location Home or Overseas
- **ii** their own Mean Kinship band and
- **iii** the Kinship Coefficient of the progeny of mating with the named mare. (This is not the same as the Mean Kinship, but is closely related. It is in-fact the inbreeding coefficient of any progeny of mating this mare and this stallion).

SELECTION

Following discussions with breeders and with staff at the Rare Breeds Survival Trust, a new "Traffic Light" was introduced in 2018, making interpretation of the tables clear and straightforward. All possible matings are ranked into one of four tiers and colour coded (Tier 1 represents the best genetic pairings whilst Tier 4 is the worst for the genetic health of the whole population).

- **TIER 1:** Mare and Stallion from the same or an adjacent Kinship Band **AND** mating resulting in progeny of lower Kinship Coefficient than the Mean Kinship of the Mare. MATINGS THAT MEET THE ABOVE REQUIREMENTS ARE SAID TO BE SPARKS COMPLIANT AND ARE HIGHLIGHTED IN GREEN ON THE DATA SHEETS. **THESE MATINGS ARE ENCOURAGED**.
- **TIER 2:** Mare and Stallion from the same or an adjacent kinship band **BUT** mating resulting in progeny of higher Kinship Coefficient than the Mare but less than 0.24. These matings represent **"The Best of the Rest"** and are the preferred alternative if a SPARKS compliant mating does not exist or is not possible. They are highlighted in yellow on the sheets.
- **TIER 3**: Mare and Stallion from widely differing Kinship Band and Kinship Coefficient less than 0.24. These matings bring together more common alleles (genes) with those that are less well represented in the population, in a way that makes it hard to separate, putting the less frequent allele at greater risk of loss in future generations. **THESE MATINGS ARE DISCOURAGED** and are highlighted in Orange on the sheets.
- **TIER 4**: Any matings producing progeny with **KINSHIP COEFFICIENTS OF 0.24 OR GREATER SHOULD BE AVOIDED.** They are Highly Inbred and increase the probability of deleterious traits being expressed in future generations. (A Kinship Coefficient of 0.25 IS EQUIVALENT TO A FULL SIBLING BROTHER / SISTER MATING!) These matings are highlighted in Red on the sheets.

CLEVELAND BAY HORSE SOCIETY - 2021

Guidance on the use of SPARKS Data Sheets

It should be noted that the "tiering" is an assessment of the merit of pairing of two animals and is not an endorsement or criticism of the genetic makeup or quality of either of the individuals.

THE AIMS AND SUCCESS OF THE PROJECT

- The retention and maximising of genetic diversity of the whole population
- The reduction of the rate of increase in inbreeding and maintaining it at a minimum (zero is not possible)
- The avoidance of mixing more common alleles with less common ones (mixing of rare bloodlines with more common ones)
- The direct effect of achieving the above will be maximising the Effective Population Size, which is a well-accepted measure of the genetic health of any population.
- Information will be freely distributed on an annual basis to enable breeders to work towards these goals.
- By adopting these strategies, we hope to prevent the loss of genetic diversity and increasing levels of inbreeding that are inherent problems in rare breeds. This in turn should help avoid future problems of increasing infertility, foal mortality, deformity and other genetically linked phenomena.

ARTIFICIAL INSEMINATION

Feedback from breeders has highlighted the need for information on the availability of stallions by artificial insemination. We have tried to indicate whether a stallion is available by fresh or frozen semen, and which countries frozen semen is available for export to. Contact details for stallion owners can be found in current Society publications or on the CBHS website http://www.clevelandbay.com/stallions .

The datasheets contain Kinship data relating to some stallions that are now deceased, that are known to or may have frozen semen still available. This information is provided so breeders have the broadest possible picture of mating choices. Please note that appearance of the existence of stored semen on the sheets is no guarantee of availability.

MEAN KINSHIP OF PROGENY

The use of datasheets since 2004 has highlighted the subtle difference between Mean Kinship of parents and Kinship Coefficient of progeny. Until new animals are registered and entered onto the system we cannot calculate their Mean Kinship or Kinship Band. This highlights the importance of prompt registration of pure-bred foals. Owners are advised to follow the above guidelines in making breeding choices, as they continue to be the most robust and scientifically validated method of ensuring the genetic wellbeing of the global Cleveland Bay population.

ANOMALIES

If when studying the datasheets for your mares you recognize information that is not or might not be correct, please let the Society know. In past years a small number of cases have come to light which revealed inconsistencies in the SPARKS data, which have since been corrected. The programme can only be as good as the data it contains, so please let us know if, for example, mares with identical breeding do not have identical datasheets.

NB NO HBLB GRANTS WILL BE PAID FOR TIER 4 (RED) HIGHLY INBRED MATINGS

The data provided in this set of sheets is highly filtered from the SPARKS database and should not be taken as a true representation of the current Cleveland Bay population. As such it is not suitable for research purposes.

Andy Dell. Endmoor. January 2021
Email andy.dell@btinternet.com

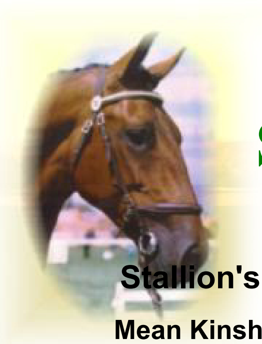

Cleveland Bay Horse Society

SPARKS For Licensed Stallions 2021

For Pure-bred Mares / Stallions Licensed For Pure Bred Breeding

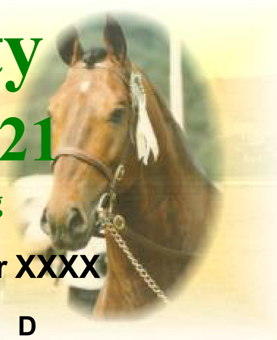

Stallion's Name **XXXXX** **Stud Book Number XXXX**

Mean Kinship 0.2119 Inbreeding Coefficient .2082 Mean Kinship Band D

NB THIS SHEET AND THE DATA IT CONTAINS IS ONLY VALID FOR THE 2021 BREEDING SEASON

Please follow the traffic lights & use in conjunction with the accompanying guidance notes

Tier 1 Matings highlighted in GREEN are SPARKS compliant & are ENCOURAGED

Tier 2 Matings highlighted in YELLOW are not fully compliant but are the "BEST OF THE REST"

Tier 3 Matings highlighted in ORANGE are not compliant AND jump Bands which is DISCOURAGED

Tier 4 Matings highlighted in RED should be AVOIDED as they are highly inbred (> 0.24)

Mare	Studbook Number	Mare's Year of Birth	Mare's Age	Mare's Pure-Bred Progeny	Mare's Inbreeding Coefficient	Mare's Mean Kinship	Kinship Band	Inbreeding Coefficient of Progeny
Aderlaides Dancing Matilda	2421	2002	19	9	.195	0.2276	E	.2
Afondale High Heaven	2483	2006	15		.2214	0.2270	E	.2003
Afondale High Hopes	2399	2004	17		.2435	0.2316	F	.2013
Afondale Taran	2522	2008	13	5	.2273	0.2290	E	.2098
Alabai Rafaela	232AUS	2010	11		.2094	0.2251	E	.2064
Annella	2336	1997	24	6	.2032	0.2206	E	.2065
Antonia	2337	1998	23		.2436	0.2310	F	.2182
Arena Moonshadow	2677	2019	2		.2293	0.2283	E	.2151
Arena Moonstone	2569	2011	10	3	.2114	0.2299	E	.2071
Arena Unique	2637	2017	4		.2014	0.2195	D	.2057
Arfon Poppet	2296	1999	22		.2149	0.2181	D	.2113
Arkland Misty Law	2459	2005	16		.2215	0.2278	E	.2094
Austral Park Brittany	217AUS	1999	22		.2728	0.2215	E	.1996
Australpark Aurora	219AUS	1998	23		.2459	0.2249	E	.2025
Bankfields Ruby	GRB237	2012	9	2	.1191	0.1763	A	.1598
Bankfields Sweet Candy	2567	2011	10		.2319	0.2349	F	.2059
Barbarian Calipso	2464	2006	15	2	.216	0.2318	F	.2156
Barbarian Divinity	2497	2007	14		.2251	0.2342	F	.2114
Barbarian Dynamic	2496	2007	14		.216	0.2316	F	.2156
Barbarian Illusion	2617	2015	6		.2005	0.2251	E	.2042
Bayhill Electra	242USA	2013	8		.2201	0.2244	E	.2144
Bcf Eowin	309USA	2011	10	2	.2055	0.2194	D	.227
BCF Ginger	305USA	2012	9		.1988	0.2185	D	.2262
Bcf Sage	273USA	2009	12		.2055	0.2193	D	.227
Bcf Visa	275USA	2012	9		.1942	0.2160	D	.2257
Beamish Empress	2325	2000	21		.2554	0.2362	F	.2162
Beamish Midnight Rose	2529	2008	13	3	.243	0.2362	F	.2124
Bearpaw Martha Jane	264USA	2015	6		.2216	0.2248	E	.2198
Belladonna Mahogany	159USA	2006	15	2	.219	0.2236	E	.2088
Belladonna Nexus	160USA	2006	15		.1853	0.2034	C	.2096
Belladonna Xtravagance	208USA	2009	12	4	.1778	0.2134	D	.2021
Bellavista Her Ryl Highnes	315USA	2019	2		.2016	0.2206	E	.2077
Belliff Rockin Ruby	222USA	2007	14		.2129	0.2206	E	.2157
Benlock Regal	2638	2017	4		.229	0.2343	F	.2183
Bensington Peggy	2275	1998	23	2	.2452	0.2441	G	.2076

NB THIS SHEET AND THE DATA IT CONTAINS IS ONLY VALID FOR THE 2021 BREEDING SEASON

Beybreeze Nadia	148USA	2000	21		.2121	0.2215	E	.1987
Billara Alice	146AUS	1996	25		.286	0.2263	E	.2052
Blackbrook Amethyst	2546	2009	12		.2158	0.2270	E	.2057
Blackbrook Sapphire	2465	2006	15		.2522	0.2250	E	.2049
Blackclough Kilclara	2476	2006	15	2	.2365	0.2369	F	.2075
Blackclough Killnicko	2596	2013	8	1	.192	0.2240	E	.2045
Blackclough Kilteely	2620	2015	6		.2043	0.2311	F	.2056
Blakeside Freya	2537	2009	12		.2082	0.2278	E	.2035
Blithfield First Edition	2633	2016	5		.2308	0.2339	F	.2098
Blithfield Midnight Rose	GRA211	2018	3		0	0.1195	A	.1067
Blithfield Miss Markle	GRB238	2018	3		.1298	0.1795	A	.1603
Blithfield My Fair Lady	2661	2018	3		.2118	0.2264	E	.2091
Borderfame Supernova	2223	1997	24		.2225	0.2358	F	.202
Botton Grove Gaelle	2534	2008	13		.2276	0.2378	F	.2023
Brackenbrae Crystal	2549	2009	12	1	.2132	0.2327	F	.2083
Brackenbrae Winnie	2686	2020	1		.2397	0.2313	F	.2243
Braelea Park Wilful	110AUS	1999	22	1	.2217	0.2267	E	.2123
Braithwaite Cari	2543	2009	12	3	.238	0.2266	E	.2446
Braithwaite Desire	2495	2007	14	1	.2425	0.2359	F	.2235
Braithwaite Eclipse	2683	2020	1		.2297	0.2349	F	.2169
Braithwaite Elise	2524	2008	13	2	.2313	0.2374	F	.2101
Braithwaite Empress	2616	2015	6		.227	0.2319	F	.2185
Braithwaite Giulietta	2663	2019	2		.2376	0.2376	F	.2125
Braithwaite Infiniti	2666	2019	2		.2247	0.2331	F	.2217
Braithwaite Ioniq Royale	2662	2019	2		.2162	0.2276	E	.2224
Braithwaite Jazz	2588	2012	9		.2337	0.2250	E	.2477
Braithwaite Liberty	2668	2019	2		.2392	0.2359	F	.2151
Braithwaite Mayflower	2564	2010	11	2	.2117	0.2267	E	.2343
Braithwaite Mulsanne	2684	2020	1		.2162	0.2276	E	.2224
Braithwaite Octavia	2397	2003	18	5	.2425	0.2365	F	.2235
Braithwaite Princess	2523	2008	13		.23	0.2255	E	.2389
Braithwaite Serena	2578	2011	10	2	.238	0.2264	E	.2446
Burfield Briar Rose	2539	2009	12	1	.2185	0.2267	E	.2285
Burfield Fern	2604	2013	8	2	.215	0.2263	E	.2102
Burfield Ivy	2687	2020	1		.2208	0.2312	F	.2084
Carlton Surprise	2332	2000	21		.2554	0.2350	F	.2107
Castlerise Suelea	2671	2019	2		.2248	0.2289	E	.2111
Castlerise Winnoa	2646	2018	3		.2217	0.2295	E	.203
Chakola's In Style	190USA	2008	13		.1898	0.2054	C	.1944
Chakolas Gala Performance	164USA	2006	15		.1898	0.2054	C	.1944
Charmed Sweet Forest	2400	1997	24		.2072	0.2296	E	.2076
Chelsea Joie de Vivre	313USA	2019	2		.2138	0.2251	E	.2049
Childrey Chez Black	2482	2006	15	4	.1909	0.2146	D	.2291
Chinashop GJ	2353	2002	19		.2623	0.2300	F	.2002
Cholderton Bellona	2293	1998	23	8	.2351	0.2328	F	.2405
Cholderton Cassandra	2284	1999	22	2		-1.0000	Not	.253
Cholderton Circe	2285	1999	22	3	.2082	0.2084	C	.2212
Cholderton Cybele	2287	1999	22	7	.2196	0.2188	D	.2442
Cholderton Danae	2318	2000	21	1		-1.0000	Not	.2802
Cholderton Demeter	2319	2000	21	1	.2099	0.2194	D	.2329
Cholderton Dido	2320	2000	21	1	.2109	0.2190	D	.2689
Cholderton Dione	2321	2000	21		.253	0.2326	F	.227
Cholderton Fable	2396	2002	19		.3173	0.2286	E	.2478
Cholderton Galadrial	2444	2003	18		.3107	0.2328	F	.2338
Cholderton Icon	2474	2006	15	6	.2081	0.2067	C	.2455
Cholderton Ida	2480	2006	15	1	.2319	0.2183	D	.2439

NB THIS SHEET AND THE DATA IT CONTAINS IS ONLY VALID FOR THE 2021 BREEDING SEASON

Stallion ***XXXXXX*** ***Stud Book Number*** ***XXXX*** ***Page No.2***

Cholderton Io	2479	2006	15	2	.2353	0.2142	D	.2518
Cholderton Kelosia	2565	2010	11	3	.3134	0.2118	D	.2362
Cholderton Nereid	2605	2013	8	2	.2108	0.2125	D	.4068
Cholderton Psyche	2625	2015	6	1	.1909	0.2144	D	.229
Cholderton Pythia	2623	2015	6	1	.2151	0.2066	C	.2358
Cholderton Rhea	2641	2017	4		.2513	0.2138	D	.268
Cholderton Selene	2659	2018	3		.2229	0.2127	D	.2721
Cholderton Theros	2673	2019	2		.2229	0.2127	D	.2721
Cholderton Thetis	2664	2019	2		.2173	0.2113	D	.2726
Cholderton Ulalia	2691	2020	1		.2082	0.2113	D	.4034
Cholderton Unice	2692	2020	1		.2065	0.2083	C	.3179
Cholderton Urania	2690	2020	1		.2173	0.2113	D	.2726
Cholderton Zinnia	2222	1996	25	2		-1.0000	Not	.2423
Cobblehills Jacqueline	167USA	2006	15		.2168	0.2279	E	.2048
Cobblehills Miranda	182USA	2007	14	3	.2019	0.2244	E	.2038
Cobblehills Paloma	183USA	2007	14		.2168	0.2279	E	.2048
Cornish (USA) Minnow	270USA	2012	9	2	.2167	0.2242	E	.2004
Cornish (USA) Myriah	266USA	2015	6		.2167	0.2241	E	.2004
Cornish (USA) Pride	269USA	2011	10		.2031	0.2199	D	.1992
Craigiewood Mull	2475	2006	15	1	.2251	0.2321	F	.2071
Craigiewood Taransay	2572	2011	10		.2267	0.2298	E	.206
Darren Queen Of Sheba	2382	2003	18		.2043	0.2272	E	.211
Dawn	2198	1996	25		.1826	0.2201	E	.198
Ditchley Lady Georgina	2632	2016	5		.2212	0.2326	F	.2066
Duchess of Malfi	2594	2013	8	1	.2344	0.2346	F	.2099
Earlswood Fedora	2499	2007	14		.2189	0.2263	E	.2179
Earlswood Fourth Edition	2259	1998	23		.233	0.2221	E	.2052
Eden Valley Illithia	2378	2003	18	2	.2194	0.2362	F	.2137
Edenvalley Wallflower	2306	2000	21	3	.2154	0.2289	E	.2091
Elemental Sunshine Hattie	2510	2007	14		.2055	0.2290	E	.2188
Embleton Harmony	2536	2008	13	1	.2651	0.2334	F	.2087
Embleton Reel	2681	2020	1		.2134	0.2302	F	.2046
Embleton Tutti	2576	2011	10	1	.2272	0.2350	F	.2013
Epiphanybay Adelyne	255USA	2015	6	1	.1981	0.2285	E	.2062
Epiphanybay Constance	206USA	2009	12	1	.1969	0.2294	E	.2042
Epiphanybay Lady Arabella	254USA	2015	6	1	.2172	0.2286	E	.2046
Epiphanybay Lady Divine Grace	317USA	2020	1		.2155	0.2279	E	.2118
Ferndale Springs Tessarae	258AUS	2013	8		.2262	0.2284	E	.2209
Ferndale Springs Topsy	215AUS	2008	13		.2347	0.2300	F	.2181
Ferndalesprings Margaret	261AUS	2013	8		.2269	0.2266	E	.2108
Ferndalesprings Shamrock	260AUS	2015	6		.2309	0.2282	E	.218
Folds Alexander	2458	2005	16	1	.2001	0.2158	D	.2401
Folds Pearl	2512	2007	14	2	.2331	0.2301	F	.2207
Forest Fair Anna	2331	2000	21		.2204	0.2272	E	.2218
Forest Fancy Girl	2343	2001	20	1	.2286	0.2305	F	.2145
Forest Favourite Girl	2392	2003	18	4	.2113	0.2363	F	.2107
Forion Gwenno	2215	1996	25		.2105	0.2325	F	.2019
Foxglove Jasmine	2542	2009	12	1	.2175	0.2197	D	.239
Foxhollow (USA) Seashell	129USA	2003	18	1	.2263	0.2160	D	.1992
Foxhollow Chesapeake	124USA	2001	20		.2457	0.2281	E	.2045
Foxhollow Regatta	105USA	1999	22		.2263	0.2159	D	.1992
Foxhollow USA Seabreeze	177USA	2007	14		.2263	0.2159	D	.1992
Foxwood Iris	2277	1998	23	2	.2193	0.2278	E	.2037
Fryup Dawn	2425	2002	19	2	.2021	0.2234	E	.2066
Fryup Thelma	2344	2000	21		.3164	0.2320	F	.2011
Fryup Tiffany	2238	1997	24	3	.2641	0.2259	E	.2059

NB THIS SHEET AND THE DATA IT CONTAINS IS ONLY VALID FOR THE 2021 BREEDING SEASON

Stallion XXXXXXXX *Stud Book Number XXXX*

SPARKS Stallion sheets 2021 V1c published on behalf of The Cleveland Bay Horse Society by Andy Dell 14/07/2021
